# Supplementary figures and images for: Development and piloting of a highly tailored digital intervention to support adherence to antihypertensive medications as an adjunct to primary care consultations
Source: BMJ Open. 2019 Jan 6;9(1):e024121. doi: 10.1136/bmjopen-2018-024121 (PMC6326276; doi:10.1136/bmjopen-2018-024121)

## Appendix 1. Intervention development process, informed by the MRC Framework.

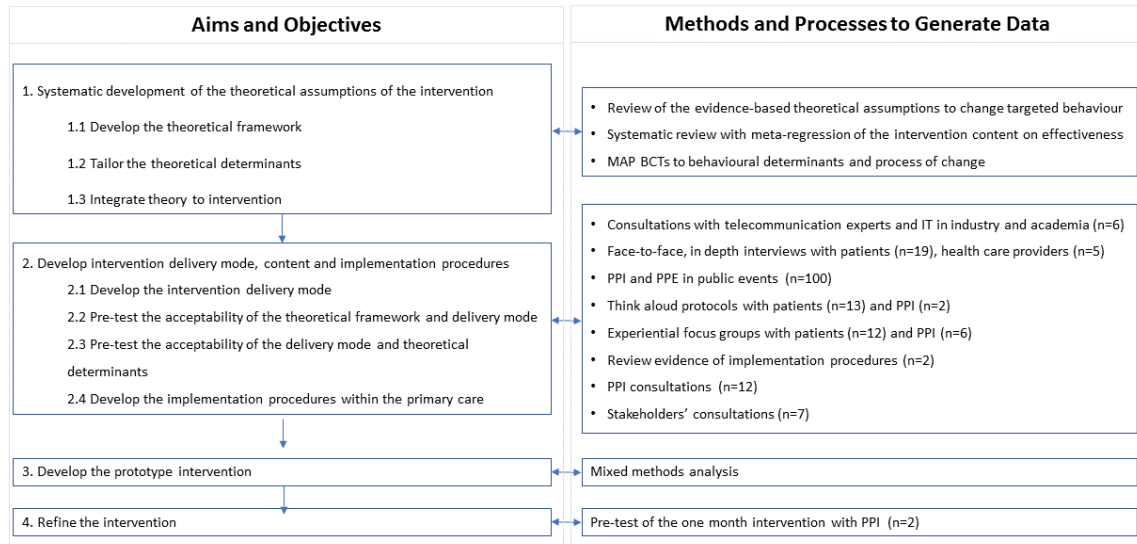

Supplement: Supplementary data [file bmjopen-2018-024121supp001.pdf]

## Appendix 2. Intervention piloting process, informed by the MRC framework.

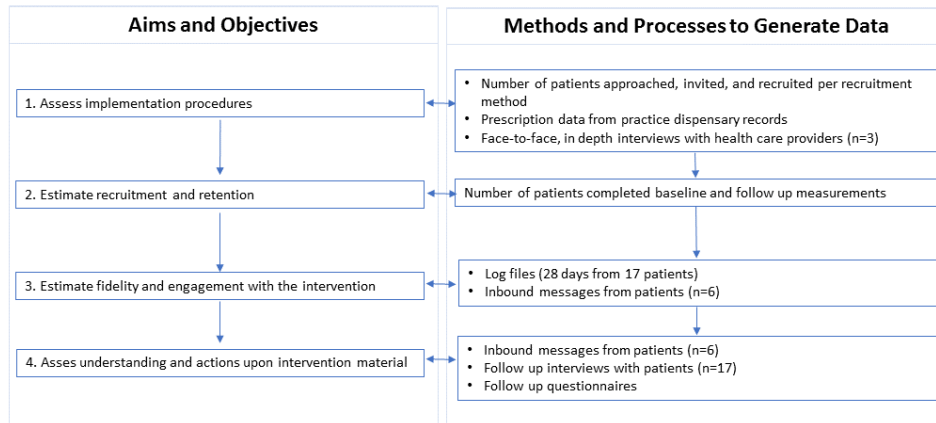

Supplement: Supplementary data [file bmjopen-2018-024121supp002.pdf]
