# Supplementary material for: Development and piloting of a highly tailored digital intervention to support adherence to antihypertensive medications as an adjunct to primary care consultations
Source: BMJ Open. 2019 Jan 6;9(1):e024121. doi: 10.1136/bmjopen-2018-024121 (PMC6326276; doi:10.1136/bmjopen-2018-024121)
Supplement: Supplementary data [file bmjopen-2018-024121supp003.pdf]

Appendix 3. Map of the BCTs to theoretical determinants and framework.

	Framework	Theoretical Determinant	BCTs
PRIMARY DETERMINANTS	INA	Beliefs and knowledge	*Re-attribution*
			Pros and cons
			Information about antecedents
			social comparison
			reduce negative emotions
			normative information
		outcome expectations	*Information about health consequences*
			information about emotional consequences
			Information about social and environmental consequences
			Pros and cons
			goal setting (behaviour)
			goal setting (outcome)
			self talk
			Social support (unspecified)
			Social support (practical)
			Socia support (emotional)
		self efficacy	*feedback on behaviour*
			reporting whether or not the behaviour was performed
			Problem solving
			action planning/ implementation intentions
			self monitoring of behaviour
			adding objects to the environment
			feedback on outcomes of behaviour
			biofeedback
			review behaviour goals
	NINA		*review outcome goals*
			feedback on emotional state
			monitoring of emotional consequences
			social reward
			Social support (unspecified)
			Social support (practical)
			Socia support (emotional)
			self talk
		habit	*Prompts/cues*
			habit formation
			action planning/ implementation intentions
			feedback on behaviour
			Social reward
SECONDARY DETERMINANTS		social support	*social comparison*
			social reward
			Social support (unspecified)
			Social support (practical)
			Socia support (emotional)
		emotional/ affective state	*Information about emotional consequences*
			Report emotional state
			Feedback on emotional state
			Monitoring of emotional consequences
