# Supplementary material for: Development and piloting of a highly tailored digital intervention to support adherence to antihypertensive medications as an adjunct to primary care consultations
Source: BMJ Open. 2019 Jan 6;9(1):e024121. doi: 10.1136/bmjopen-2018-024121 (PMC6326276; doi:10.1136/bmjopen-2018-024121)
Supplement: Supplementary data [file bmjopen-2018-024121supp004.pdf]

**Appendix 4**. Examples of Patient and Public Involvement and Engagement (PPI/E) messages.

| **Scenario:** Mark is a 49 year old car mechanic. He was diagnosed with high blood pressure by his GP 6 months ago even though he had no symptoms, was not overweight, did not smoke, ate healthily and had an active job. Initially, he took the medication exactly as his GP prescribed every day. However, after a few weeks he began to experience constipation and read on the leaflet supplied with the tablets that this was a side effect caused by the medication. Now, he only takes his medication every other day and the constipation has cleared up. However, his blood pressure is still too high. | |
|---|---|
| **Message** | **Event** |
| Keep on the tablets prescribed to you by your Doctor. High blood pressure is more serious than constipation. | HOP |
| His action was foolish – he should have consulted his GP before changing the prescribed medication. | HOP |
| This scenario similar to mine. However I do take my meds without fail. I am motivated by my family history which leads me to believe I may be susceptible to heart disease which, of course, is thought to be related to blood pressure. I can understand why people might be concerned about side effects, so they need motivation. | HOP |
| I know that mechanics are frequently asked to work through lunch breaks etc. Please take some fruit and water to work with you to snack on throughout the day. This will help with your constipation, as you should be taking your pills every day. Good luck. | HOP |
| You need to talk to your GP in order for him to prescribe a laxative to help with the constipation. The need to take your BP medication is crucial in bringing your BP down. | HOP |
| BP tablets must be taken every day so consult your GP for medication for constipation. | HOP |
| It’s stupid not to take your meds! If you’re constipated just eat more fibre. It’s not worth the risk. | HOP |
| Hey Mark. Are you aware of your blood pressure today? Have you taken your meds yet? | SF |
| Hi Mark. I sympathise a lot. I used to suffer from blood pressure and constipation, but nowadays I rarely suffer from either. I’ve had heart surgery but still cycle a lot. Don’t give up on the meds. There are different tablets available: ask your GP. But also, use whole brand cereals regularly, even at bedtime! | SF |
| Hi Mark. You’ve sorted the constipation – keep going for the blood pressure! | SF |
| Mark, you should keep taking your medication even with the side effects, because it is better for you. | SF |

Abbreviations: Hospital Open Event – HOP; Science Festival – SF; Focus Groups – FG
