# Supplementary material for: Development and piloting of a highly tailored digital intervention to support adherence to antihypertensive medications as an adjunct to primary care consultations
Source: BMJ Open. 2019 Jan 6;9(1):e024121. doi: 10.1136/bmjopen-2018-024121 (PMC6326276; doi:10.1136/bmjopen-2018-024121)
Supplement: Supplementary data [file bmjopen-2018-024121supp005.pdf]

Appendix 5. Examples of tailoring messages.

Tailored theoretical determinants	BCT	Message file
↑ NINA and ↑ emotional state	Habit formation	Please do not forget to take your tablets. To achieve better control over high blood pressure, you will need to take them every day.
NINA / INA and ↓ social norms	Report whether or not the behaviour was performed, social reward, and information about health consequences	IVR: We are calling to see how you are doing with taking your tablets. Have you taken all your tablets as prescribed today?<br>P: yes<br>IVR: Well done! Taking your medications as prescribed is an easy way to keep blood pressure down.
↑ NINA	Habit formation	You are soon due to take your tablet. Please do not forget to do so.
NINA / INA and prescription plan	Habit formation and action planning	This is your message for your blood pressure tablets. One easy way to remember your tablets, is to take them with another daily activity, such as your morning cup of tea. If they are always done together, it will be harder to forget.
↑ Beliefs about medications, ↑ self-efficacy to take medications when no health benefits and ↑ emotional state	Habit formation and information about emotional consequences	Please keep taking your tablets as prescribed even if you are well and feeling healthy. High blood pressure is one of those things that unless you actually feel it you're not aware that it is a problem. Taking your medications as prescribed will support you to keep enjoying things or activities that are important to you.
NINA, ↓ beliefs about medications and ↓ emotional state	Habit formation and social support (unspecified)	Whatever the day may holds, please do not forget to take your tablets. To achieve better control over high blood pressure, you will need to take them every day.
↓ self-efficacy to cope with ↓ practical social support	Habit formation and social support (unspecified)	We are calling to see how you are doing with taking your tablets today. Please remember that you can always call the free telephone number if you require more support on how to take your tablets as prescribed. If you require more information about the health condition or the dosage of your medications, please contact your GP practice or pharmacist.
↑ social norms and ↑ self-efficacy to cope with ↓ practical social support	Habit formation and social support (unspecified)	You may find it helpful to ask people who are important to you to remind you to take your medications as prescribed and maintain your blood pressure under control. Please remember that you can always call the free telephone number if you require more support on how to take your tablets as prescribed.

Note: ↑indicate higher score from the cut-off point, ↓ indicate lower score from cut-off point.
