# Supplementary material for: Development and piloting of a highly tailored digital intervention to support adherence to antihypertensive medications as an adjunct to primary care consultations
Source: BMJ Open. 2019 Jan 6;9(1):e024121. doi: 10.1136/bmjopen-2018-024121 (PMC6326276; doi:10.1136/bmjopen-2018-024121)
Supplement: Supplementary data [file bmjopen-2018-024121supp006.pdf]

Appendix 6. Participants' demographic characteristics

Age		Work status		Health condition		Gender	
80-89	7.8%	Retired	76.5%	Hypertension	64%	Male	65.7%
70-79	28.1%	Part-time	10.9%	Type 2 Diabetes	14%	Female	34.3%
60-69	43.7%	Full time	10.9%	Co-morbidities	22%		
50-29	15.6%	Unable to work	1.7%				
40-49	4.6%						

Note: results from N=64 patients taking part in intervention development studies (interviews n=19; think aloud protocols n=13; focus groups n=12; consultations n=2) and pilot study (n=18).
