# Supplementary material for: Development and piloting of a highly tailored digital intervention to support adherence to antihypertensive medications as an adjunct to primary care consultations
Source: BMJ Open. 2019 Jan 6;9(1):e024121. doi: 10.1136/bmjopen-2018-024121 (PMC6326276; doi:10.1136/bmjopen-2018-024121)
Supplement: Supplementary data [file bmjopen-2018-024121supp007.pdf]

Appendix 7. Participants' perception of the intervention utility and future use

	Mean (sd)
The intervention messages helped me to take my tablets as prescribed	3.2 (1.3)
The intervention reminded me to take my medications as prescribed	3.5 (1.3)
The intervention was easy to use	4.3 (0.7)
The intervention addressed my personal needs	3.3 (1.1)
It would have been useful, if the intervention was part of the regular care	3.7 (0.9)
I would recommend the intervention to other people who take medications for long-term health conditions	4.2 (0.8)

Note: Likert scale, 1 strongly disagree to 5 strongly agree. Data from n=17 participants.
