# Supplementary material for: Development and piloting of a highly tailored digital intervention to support adherence to antihypertensive medications as an adjunct to primary care consultations
Source: BMJ Open. 2019 Jan 6;9(1):e024121. doi: 10.1136/bmjopen-2018-024121 (PMC6326276; doi:10.1136/bmjopen-2018-024121)
Supplement: Supplementary data [file bmjopen-2018-024121supp008.pdf]

Appendix 8. Participants' satisfaction with the intervention components.

	Mean (sd)
The experience with the intervention	4 (0.7)
The voice delivering the intervention messages	3.7 (1.1)
The content of the intervention messages	3.8 (0.8)
Being able to call the intervention	4 (0.7)
The availability of the intervention 24/7	4.2 (0.8)
The response you received from the intervention	3.4 (0.7)
The response you received to your particular queries/questions	3.4 (0.7)

Note: Likert scale, 1 very dissatisfied to 5 very satisfied. Data from n=17 participants.
