# Supplementary material for: Development and piloting of a highly tailored digital intervention to support adherence to antihypertensive medications as an adjunct to primary care consultations
Source: BMJ Open. 2019 Jan 6;9(1):e024121. doi: 10.1136/bmjopen-2018-024121 (PMC6326276; doi:10.1136/bmjopen-2018-024121)
Supplement: Author's manuscript [file bmjopen-2018-024121.draft_revisions.pdf]

BMJ Open is committed to open peer review. As part of this commitment we make the peer review history of every article we publish publicly available.

When an article is published we post the peer reviewers' comments and the authors' responses online. We also post the versions of the paper that were used during peer review. These are the versions that the peer review comments apply to.

The versions of the paper that follow are the versions that were submitted during the peer review process. They are not the versions of record or the final published versions. They should not be cited or distributed as the published version of this manuscript.

BMJ Open is an open access journal and the full, final, typeset and author-corrected version of record of the manuscript is available on our site with no access controls, subscription charges or pay-per-view fees (http://bmjopen.bmj.com).

If you have any questions on BMJ Open's open peer review process please email info.bmjopen@bmj.com

BMJ Open

Development and piloting of a highly tailored text and voice messaging intervention to support adherence to antihypertensive medications within the primary care setting

Journal:	*BMJ Open*
Manuscript ID	bmjopen-2018-024121
Article Type:	Research
Date Submitted by the Author:	14-May-2018

Complete List of Authors:	Kassavou, Katerina; University of Cambridge, Public Health and Primary Care ; University of Cambridge,<br>Houghton , Vikki<br>Edwards, Simon<br>Brimicombe, James; University of Cambridge, Primary Care Unit, Department of Public Health and Primary Care<br>Sutton, Stephen; University of Cambridge,

Keywords:	medication adherence, Hypertension < CARDIOLOGY, interactive voice response, text messages, pilot intervention

SCHOLARONE™
Manuscripts

For peer review only

INTERVENTION DEVELOPMENT AND PILOTING

Development and piloting of a highly tailored text and voice messaging intervention to support adherence to antihypertensive medications within the primary care setting

Aikaterini Kassavou, Vikki Houghton, Simon Edwards, James Brimicombe, and Stephen Sutton

Address: University of Cambridge, School of Clinical Medicine, Department of Public Health and Primary Care, Forvie Site, Robinson Way, Cambridge, CB2 0SR

Corresponding author: Aikaterini Kassavou, University of Cambridge, School of Clinical Medicine, Department of Public Health and Primary Care, Forvie Site, Robinson Way, Cambridge, CB2 0SR.

Key words: medication adherence, hypertension, interactive voice response, text messages.

Word count: 4878

For peer review only

Abstract

Objectives: This paper describes the systematic development and piloting of a highly tailored text and voice message intervention to increase adherence to medication within primary care.

Methods: following the MRC guidance this paper describes: a) the systematic development of the theoretical framework, based on review of theories and meta-analyses of effectiveness; b) the systematic development of the delivery mode, intervention content and implementation procedures, based on consultations, face-to-face interviews, think-aloud protocols, focus groups, systematic reviews, PPI/E input, intervention pre-test, with c) the piloting of the intervention, based on a one-month intervention; and follow up assessment including interviews and questionnaires. The mixed methods analysis combined findings from the parallel studies complementarily.

Results: intervention development suggested the target behaviour of the intervention should be the tablets taken at a regular time of the day. It recommended that participants could be more receptive to intervention content when they initiate medication taking or they change prescription plan; and more emphasis is needed to patients' consent process. Intervention piloting found high intervention engagement with, and fidelity of, the intervention content; which included a combination of BCTs, and was highly tailored to patients' beliefs, and prescription plan. Participants reported that the intervention content increased awareness about the necessity to take and maintain adherent to medication, reinforced social support and habit formation, and reminded them to take medication as prescribed.

Conclusion: tailored automated text and voice message interventions are feasible ways to increase medication adherence, as an adjunct to primary care.

For peer review only - http://bmjopen.bmj.com/site/about/guidelines.xhtml

Keywords: adherence, anti-hypertensive medication, text messaging, interactive voice response.

Strengths and limitations of this study

- This is the first medication adherence intervention for patients with hypertension and co morbidities that has been developed and piloted within the UK primary care setting
- The study used rigorous data collection methods and analysis, from multiple perspectives
- This study involved and engaged Patients and Public in all stages of the development and piloting of the intervention
- Future studies should include a real time measurement of the behaviour and provide tailored feedback
- Future studies should include objective measures of the intervention determinants and outcomes.

For peer review only

Introduction

Hypertension or high blood pressure (HBP), affects approximately one in four people in England [1], and often accompanies other conditions, including type 2 diabetes mellitus (T2DM), coronary heart disease and stroke [2]. These conditions are major risk factors for disability and premature death [2], and medication adherence can significantly lower these risks [1]. For example, in England over ten years, an estimated 7,000 quality of life years could be saved and £120m not spent on related health and social care, if people had better adherence to anti-hypertensive medications [3].

Adherence is defined as taking at least 80% of the prescribed tablets, and/or having medications dispensed for at least 95% days of a prescribed period [4]. However, substantial proportions of patients do not take their medication as prescribed [5]. A recent meta-analysis showed that 40% of people do not adhere adequately to cardiovascular medication and the percentage was similar (41%) for non-adherence to anti-hypertensive medications [6]. Taking into consideration the growing prevalence of HBP, co-morbidities and the ageing population [7], especially in lower and middle-income countries [8], it is likely that there will be an increase in demand for health care resources to support medication adherence. NICE recommends the development of novel and cost-effective interventions to aid patient's decision making about taking medicines, as an adjunct to health care providers' consultations [9, 10].

Tailored mHealth interventions, such as text (SMS) and voice (IVR) messaging intervention, is one way to support patients' adherence between their consultations. Review evidence found that such interventions can effectively support adherence to different types of medication, including antihypertensive tablets, when compared to usual care only [11, 12], and can potentially be cost-effective if applied to large number of people [13]. Evidence suggest that there is a scope of developing tailored interventions, since these seem to be

For peer review only - http://bmjopen.bmj.com/site/about/guidelines.xhtml

more effective than non-tailored interventions [14]. However, no such intervention has been developed and piloted in the UK.

The aim of this paper is to describe the development and piloting of a highly tailored text and voice messaging intervention to support adherence to antihypertensive tablets. Although several guidelines have been proposed to the development of mHealth interventions [15-17], this paper is informed by the MRC Framework [18] as it leads not only the process to develop the intervention content and delivery mode, but also the implementation procedures within the primary care National Health Service (NHS), which is an important process in developing and piloting novel interventions.

Methods

Review of theory and evidence, interviews, focus groups, consultations and Patients and Public (PPI) involvement and Engagement (PPE) informed the intervention development process (see Appendix 1); and a one-month pre-post intervention study with follow up assessment informed the piloting of the intervention (see Appendix 2).

Intervention Development

Step 1. Systematic Development of the Tailored Intervention.

The systematic development of the tailored intervention was informed by the guidance provided by Dijkstra and De Vries [19] and Kreuter and colleagues [20] on the process of developing computer telephony interventions. This process included development of the theoretical framework, tailoring, and integration.

Develop the theoretical framework. The theoretical framework that distinguishes between intentional non-adherence (INA) and non-intentional non-adherence (NINA) guided the development of this intervention [21]. To address INA and NINA, we identified the empirical evidence of the proximal and modifiable determinants of adherence to medication [22] and mapped them into INA and NINA framework. After that, we used the Taxonomy of the Behaviour Change Techniques (BCTs) [23] to identify the techniques that impact on INA and NINA determinants (see Appendix 3). Our decision on the BCTs for this

For peer review only - http://bmjopen.bmj.com/site/about/guidelines.xhtml

intervention was informed by findings from two systematic literature reviews [11, 12] and previous empirical evidence [24].

Tailor the theoretical determinants. To develop a highly tailored intervention we produced a tailoring matrix, which consist of a theory-based questionnaire, an algorithm of decision rules, the schedule and the message file. The theory-based questionnaire includes items measuring each participant's values of the theoretical determinants. The algorithm includes decision rules (i.e., cut-off points), which facilitates the decisions on what message to prioritize and indicates a default feedback. The tailoring process of the intervention messages is informed by multiple sources, which can be grouped in three 'clusters': the first cluster of tailoring is based on participant's unique characteristics (e.g., name) and is informed by GP practice records; it aims to gain person's attention to the intervention by making messages personally relevant. At the second cluster of tailoring we use data from participant's responses at the tailoring questionnaire and their prescription plan. This cluster includes two sub-levels of tailoring: tailoring based on the degree of (a) INA and NINA, and (b) each of the modifiable INA and NINA theoretical determinants. At the third cluster, we tailor the intervention to participant's responses and feedback during the intervention. Participants are able to further tailor the intervention content by requesting a higher or lower degree of information for a particular determinant or by requesting feedback on a different determinant. Intervention tailoring at this cluster takes into consideration participant's navigation options until the request. The schedule includes the frequency of the messages for the duration of the intervention, which participants can increase, reduce or stop. The message file included the tailored BCTs.

Integrate theory to intervention. The frequency, combination and sequence of the BCTs were integrated into one intervention. The decision on the above elements was based on the phase participants are in (e.g., uptake or maintain), having as the starting point the collection of the medication from the dispensary.

Step 2. Development of the Intervention Delivery Mode, Content, and Implementation Procedures.

For peer review only - http://bmjopen.bmj.com/site/about/guidelines.xhtml

INTERVENTION DEVELOPMENT AND PILOTING

This step of the intervention development aimed to convert the theoretical underpinnings into features of the delivery mode, intervention content, and implementation procedures, and it involved: the development of the delivery mode, the pre-test of the theoretical framework and the delivery mode, the pre-test of the delivery mode and the theoretical determinant, and the development of the implementation procedures within the primary care.

Develop the intervention delivery mode. To translate the theoretical underpinning into features of the intervention delivery mode, consultations with six experts from the industry and academia were conducted. Meetings with telecommunication and IT experts in the University of Cambridge informed the decisions about the development of the IVR application. The discussions informed the development of features of the IVR applications, which involved the navigation options, the degree of interactivity, the voice delivering the messages, and the prompts to facilitate participants' navigation and dialogue. The discussions for the development of the SMS application involved the necessary features to accommodate custom messages on complex prescription plans. A custom written database application contained the functionality to create output for both delivery modes, and data were transferred at regular intervals to both platforms (IVR and SMS) using secure data hosting protocols. The digital platform could also facilitate participants' feedback during the intervention or at a different time (i.e., by triggering an inbound call). Voice recognition facilitated interaction and data collection. The digital platform enabled flexible schedule of messages delivery; for example, messages could be delivered at times tailored to participant's prescription plan (e.g., 1-4 messages per day).

Pre-test the acceptability of the theoretical framework and delivery mode. One interview study and Patients and Public Involvement (PPI) and Engagement (PPE) informed the acceptability of the theoretical framework and delivery mode.

Face-to-face, in depth interviews were conducted with patients and health care providers to assess the acceptability of the delivery mode, the theoretical framework, and generate relevant content. Participants were randomly identified and selected by the practice manager from practice databases. Primary care practices within the Cambridgeshire and Peterborough Clinical Commissioning Group (CCG) were eligible for inclusion, and five primary care practices from different areas of deprivation (based on Index of Multiple

For peer review only - http://bmjopen.bmj.com/site/about/guidelines.xhtml

Deprivation; IMD) took part in the study. Patients were eligible if they: (a) had a diagnosis of HBP, T2DM or both health conditions; (b) had been prescribed at least one anti-hypertensive and/or glucose lowering medication as confirmed by practice records for at least three months before recruitment; (c) had poorly controlled blood pressure or glucose levels as indicated by practice records or had gaps in collecting repeat prescriptions; and (d) were aged 40 years or older. Patients were excluded when they: (a) had hearing or speaking impairment, (b) had a diagnosis of dementia, aphasia or other cognitive difficulties that could affect the study participation, (c) had a recent severe life-threatening event, (d) had difficulty in speaking or understanding English, or (e) participated in another study. A practice GP double screened the list of selected patients against the inclusion and exclusion criteria to confirm eligibility. Eligible patients were invited by post or telephone calls following up a postage invitation. Health care providers (i.e., nurses and health care assistants) who had experience of advising patients about their medications were eligible to participate and interviewed in the practices. Nineteen patients (mean age 62.3 years old) and five health care providers were interviewed face-to-face. Both patients and health care providers' interview data informed inductively the content of the theoretical determinants and the BCTs, and provided their views on the acceptability of the automated intervention within the primary care setting.

Patients and Public Involvement (PPI) and Engagement (PPE) was conducted to assess acceptability of the delivery mode and the theoretical framework, and inform the intervention content. PPI/E members were recruited opportunistically using emails or adverts. In total 100 PPI/E members took part in three events: one meeting with a diabetes patients' group (n=20), one open event at the Addenbrookes' Hospital (n=30), and one open event at the Cambridge Science Festival (n=50). During the three PPI/E events, members were asked their views and recommendations on the delivery mode, and to write messages tailored to INA and NINA case studies. Data generated from PPI/E informed the content of the message file (for example, see Appendix 4). In overall, PPI/E members seemed to be more acceptable to NINA case studies and seemed to find it easier to generate messages for NINA rather than INA case studies.

For peer review only - http://bmjopen.bmj.com/site/about/guidelines.xhtml

Pre-test feasibility of intervention delivery mode and theoretical determinants. Four studies informed the acceptability of the delivery mode and the theoretical determinants. These included: think aloud interviews, experiential focus groups, and PPI.

Face-to-face, in depth interviews were conducted with a subsample of patients (n=13) recruited at the study mentioned above. Think aloud protocol was used to assess reliability of the mechanisms of change (i.e., link between the theoretical determinants and the BCTs), and usability of the delivery mode. All interviews were conducted at participants' places. Field notes and think aloud data informed the analysis, and results refined the tailoring of the theoretical determinants [25].

Face-to-face meetings were conducted with two PPI members recruited using emails from the list of the NIHR Cambridge BRC PPI/E Panel and Communications office. Think aloud protocol was used to assess the reliability and sequence of the items to form the measurement of the theoretical determinants (i.e., theory-based questionnaire). Both interviews were conducted at the University. Field notes and think aloud data informed the analysis, and results refined the reliability of the tailoring questionnaire and informed the decision rules of the tailoring algorithm.

Experiential focus groups aimed to obtain participants' views of, and refine, the tailored intervention content (e.g., inclusion of medication name, dose and timings; 'please do not forget to take 1 tablet ramipril 10 mg at 9am') and delivery mode within a social interaction context [26]. Eligibility criteria and recruitment procedure were similar to patients' face-to-face interviews, reported above. Four focus groups took place in primary care practices (n=2) or community centres (n=2). During the focus groups patients (n=12) received examples of SMS and IVR messages at their mobile phones and were asked to provide experiential feedback. Participants were asked their views and recommendations about the combination of the delivery modes and the intervention content, using a think-aloud protocol.

One experiential focus group with six PPI members aimed to obtain views and recommendations on the intervention content and delivery mode. PPI were recruited using emails from the NIHR Cambridge BRC PPI/E office. PPI members were eligible to take part if

For peer review only - http://bmjopen.bmj.com/site/about/guidelines.xhtml

they were prescribed medications for a long-term health condition and were over 40 years old.

The data generated from the experiential focus groups informed the content of the message file and the mapping of the intervention messages to the theoretical determinants.

Develop implementation procedures within primary care. The intervention implementation procedures were informed by systematic review evidence, PPI, and stakeholders' consultations.

Information about the recruitment setting and participants' characteristics at baseline, as well as uptake and retention rates were extracted from the two systematic reviews of trials on medication adherence delivered by SMS and/or IVR messages [11, 12]. Due to missing data, we synthesised the findings narratively. Results informed the decisions regarding the recruitment setting and methods, as well as the targeted behaviour and population.

Twelve PPI members provided feedback on the recruitment material (e.g., patients' information sheets, posters, leaflets, flyers, and invitation letters) using emails or face-to-face meetings. Their comments informed the content of the recruitment material and the description of the recruitment, informed consent, and intervention procedures in lay language.

Stakeholders' consultations were conducted with health care providers, commissioners, and patients to inform the study design and the implementation procedures within the primary care setting (e.g., recruitment, informed consent, data collection). Practitioners with experience of advising patients for medications; commissioners for medicine optimisation, prescribing or cardiovascular conditions; and patients with either HBP or both HPB and T2DM were included. Commissioners were recruited informally by email through personal networks. Patients and health care providers (i.e., practice nurses, health care assistants) were recruited through the primary care practices. Those who consented to take part were invited to the consultations. Consultation modes were flexible to maximise participation (n=3 face-to-face, n= 4 email). Stakeholders (N=7) were given a description of the proposed intervention and asked their views and recommendations. Health care providers (n=4) were consulted about the time to initiate recruitment, and the

For peer review only - http://bmjopen.bmj.com/site/about/guidelines.xhtml

recruitment methods and procedures. Commissioners (n=1) were consulted about how best to track resource use and costs and the evidence needed to inform whether or not to commission such an intervention. Patients (n= 2) were consulted about the delivery mode and the theoretical framework. All stakeholders were asked for their recommendations about the implementation procedures. The data generated from stakeholders' consultations informed the study and intervention implementation procedures.

Step 3. Develop the Prototype Intervention.

A prototype intervention was developed by multiple and iterative syntheses of the data obtained during the intervention development [27]. The intervention is highly tailored to deliver very brief (≥1 minute), theory-based messages at participant's preferred time, telephone to receive the calls (i.e., primary and/or secondary number), and at frequency of calls (e.g., repeat options). The pilot intervention consisted of 29 messages, delivered daily for a duration of one prescription-based month. Two messages were delivered at the first day of the intervention, with feedback tailored to each participant's responses at the baseline questionnaire and prescription plan, and one message per day during the following 27 days of the intervention.

Step 4. Refine the Intervention.

Two PPI members, recruited by the NIHR Cambridge BRC PPI/E office, pre-tested the prototype intervention for one prescription-based month. PPI provided their experiential feedback about the intervention usability and mechanisms of change during four, weekly telephone-based interviews with a researcher, and completed the follow up questionnaire. The data informed the decision rules of the tailoring algorithm and refined the frequency (i.e., message schedule), combination and sequence of the BCTs (i.e., integration of theory into the intervention), before piloting the prototype intervention within primary care.

Intervention Piloting

For peer review only

INTERVENTION DEVELOPMENT AND PILOTING

A one-month pilot intervention was conducted to assess (a) implementation procedures, (b) uptake, retention, fidelity, and engagement with the intervention, (c) participants' views, understanding, and actions upon the intervention content; and obtain (d) recommendations for improvement.

Patients with HBP were recruited by primary care practices within the Cambridgeshire and Peterborough CCG, and health care providers who consulted patients for taking medications. Patients were selected and recruited using the same recruitment procedure described in the intervention development studies but included two additional recruitment strategies: (a) face-to-face recruitment by health care providers, and (b) leaflets and posters in the practice waiting rooms. Health care providers received training by a researcher on the face-to-face recruitment procedures. Patients completed measures at baseline (T1) and at the end of the intervention (T2).

Patients completed the tailoring questionnaire (T1, T2), their preferred primary and secondary telephone number to receive the calls, and the time intervals of repeated calls per telephone number (T1), two items measuring adherence (T1, T2), 16 items measuring their experience and satisfaction with elements of the intervention (T2), and face-to-face interviews with a member of the research team (T2). Medication collection data for the duration of three months before T1, and at T2, were obtained from the practice dispensary records. Telephone log files assessed uptake, retention, fidelity, and engagement with the intervention. Participants' inbound calls assessed intervention engagement and impact. Face-to-face interviews (T2) assessed users' understanding and actions upon the intervention and obtained recommendations for improvement. Face-to-face interviews with health care providers (n=3) assessed implementation procedures and obtained recommendations for improvement.

Analysis

Data generated from the intervention development and pilot study were integrated into one mixed methods analysis [27], using the technique called "following a thread" [28, 29]. The theoretical underpinnings of the intervention were explored across different methods of data collections, data generated from each method informed each intervention

component (i.e., intervention content and implementation procedures) iteratively, and questions or contradictory findings generated were followed across the other methods until saturation was achieved. A summary of the outcomes of the mixed methods analysis are reported in table 1.

Table 1. Outcomes and results from intervention development and piloting

Intervention development	Intervention piloting
• Theory based questionnaire<br>• Tailoring algorithm<br>• Message file<br>• Message schedule<br>• Delivery mode<br>• Targeted behaviour and participants' characteristics<br>• Implementation procedures within the primary care	• Recruitment and retention rates<br>• Fidelity and engagement with the intervention<br>• Understanding and actions upon intervention material

For peer review only

Results

Theory Based Questionnaire

Participants' feedback on the questionnaire suggested that self-reported measures of non-adherence have confounded effect on measures of psychological constructs of adherence, and thus an objective measure of adherence was recommended. For example, Medication Adherence Report Scale items were perceived to measure INA /NINA constructs, rather than taking or not taking medications.

Tailoring

Participants' input suggested amendments to the tailoring algorithm, to increase the sensitivity of the algorithm in selecting INA participants. For example, the cut-off points of the algorithm were amended to facilitate the identification of INA and NINA. Moreover, participants' input suggested that the theoretical determinants could be the same for INA and NINA but they might differ in terms of the value of tailoring, thus we adjusted the theoretical model and cut-off points, so that each of the theoretical determinant could appear to both INA and NINA participants (for example, see Appendix 5, for examples of tailoring BCTs).

For peer review only - http://bmjopen.bmj.com/site/about/guidelines.xhtml

Message File

Participants suggested content that informed the intervention BCTs, so that we included more BCTs, such as information about social and environmental consequences.

> "*Do you, sort of, remind them of the costs and things? Is that in with medication and things, if they're missing it, or every time they lose blood pressure medication or throw it in the bin, what is the cost?*" Practitioner consultation

Schedule

The one-month intervention schedule included daily messages and participants could change the frequency or stop them. Patients at follow up interviews, recommended an intervention of longer duration with messages to gradually decrease in frequency and include advice and support for self-monitoring medication taking.

Targeted Behaviour and Participants' Characteristics

Participants reported that it was easier for them to report on the tablets or pills taken per day, rather than the specific type of tablets (e.g., felodipine, statins). However, they reported that this information could be useful for patients who initiate medication taking, and need more education about their prescribed regimens. Patients reported that the timing of medication-taking or the routine associated with the timing (e.g., lunch), rather than the type of medications to take, was easier reminder of behaviour. That was particularly important for those patients taking medication for longer and for more than one health conditions.

> Q: "*How many tablets do you take per day?*" R: "*Oh, about 17*". Q: "*How many different types of medication do you take per days?*" R: "*Yeah. That takes more counting and a bit more thinking ... I suppose, as well, if you have so many, you just get used to the times…Morning, lunch, evening, night time*" patient

Although patients with HBP, T2DM or both conditions were eligible and invited to the intervention development studies, the majority of recruited participants had primary diagnosis of HBP and reported a range of prescribed tablets (mean=6 tablets per day, sd=4.6). Therefore, the pilot intervention targeted patients with primary diagnosis of HBP.

For peer review only

Implementation Procedures within Primary Care

Health care providers who conducted the recruitment procedures, reported challenges with recruiting non-adherent patients and recommended recruitment strategies delivered at times when patients might be more receptive to uptake such an intervention. In accordance to the systematic review evidence, it was recommended that patients would be more receptive to uptake the intervention when they initiate medication taking or when there is a change to their current prescription plan (e.g., this could be either due to a health event and/or due change to the pharmaceutical product). Otherwise, patients could be reluctant to provide consent and uptake the intervention (table 2).

Table 2. Health care providers' experiential feedback

Theme	Quote
Views about recruiting non-adherent patients	*"[patients who do not adhere to medication] they're just not coming in for their monitoring … a lot of the names on the list [of eligible participants] were people that weren't coming in anyway and that was the biggest problem"* practice nurse
Views about barriers to recruit for a digital intervention	*"cause they thought it was them, they had bad blood pressure or something … there was a question about what other medication do you take as well, and they're like, 'Why do we have to answer that if it's only about the blood pressure one?' … but he felt like they were invading his space, sort of thing, by putting down all the medications, you can kind of guess what they're for, and that's what he didn't really want to"* practice nurse
Views and recommendations about recruitment methods	Annual reviews: *"annual review is a bit more time … when its annual reviews because we're actually talking about their tablets"* practice nurse recommendation about recruitment procedures<br>Practice website: *"but you can put a facility on the online forum. A lot of people access our website, so there are quite a lot of other places that people might look maybe more than a poster"* practice nurse<br>Medication prescriptions: *"but every time you get a repeat prescription list, you can write something on it"* practice nurse

Note. N=8 health care providers: nurses and health care assistants. Themes have been coded at interviews with health care providers at development (n=5) and piloting of the intervention (n=3). Quotes reported in table are from experiential interviews with health care providers (T2).

Health care providers recommended that recruitment methods could be integrated to medication annual reviews or to patients' prescriptions. They also recommended that non-adherent patients, who do not attend the practice for their medication reviews, either because they do not appreciate the need of these or they are sceptical about the

For peer review only - http://bmjopen.bmj.com/site/about/guidelines.xhtml

recommended treatment, could be more receptive to the intervention if invited by the other methods, such as text messages, newsletters, or multimedia (table 2).

However, health care providers also revealed that time constrains prevented them from explaining the implementation procedures to eligible patients during primary care consultations. In line with data from the PPI consultations and patients' interviews, health care providers suggested that there is a lot of information in the recruitment material and suggested that more emphasis is needed to patient's consent process. It was suggested that patients should be made aware about what information will be collected by their practice records, and how these will be utilised from the intervention. To facilitate patients' understanding and standardise the implementation procedures, including the informed consent, we developed videos aligned to written material, and integrated these into a brief face-to-face recruitment appointment.

To reinforce the uptake and integration of the intervention into current NHS, commissioners, patients, and health care providers reported that intervention needs to be of low or no cost. *"Would need to be very low cost or free to use"* commissioner consultation.

Intervention Uptake and Retention

Twenty participants were recruited. Eighteen provided written informed consent and registered into the study, of whom 17 completed the one-month intervention: one participant was excluded by the research team before registration because he/she was meeting one of the exclusion criteria, one participant withdrew before registration to the IVR, and the other participant dropped out during the intervention. All 17 participants completed the measures at baseline and at the end of the intervention. All primary care practices provided completed baseline (e.g., refill prescription data three months before the start of the intervention) and follow up data, for all participants.

Intervention Fidelity and Engagement

During the one-month intervention on average 29 calls were scheduled, of which 22.52 calls received by participants. On average 37 calls were made, of which 2.8 failed to be made due to technical issues and 13 calls made but failed to go through because they were not picked

For peer review only - http://bmjopen.bmj.com/site/about/guidelines.xhtml

up by the participants. On average three calls were repeated and received by participants. Ten out of the 17 participants had chosen the calls to be repeated if not answered, and five out of the 17 participants had provided a secondary number for the calls to be repeated, if there was no answered at the primary number. Overall, participants were engaged with the intervention and made inbound calls to change the schedule of the messages or report about intervention content. In summary, there was a good fidelity of the intervention BCTs, with the majority of the BCTs having high fidelity scores (table 3).

Table 3. Fidelity and engagement, intervention content

Messages, including one or more BCTs	*Scheduled*	*Received*
Tailored to baseline questionnaire, integration of tailored BCTs	1	0.6
Personalised	29	22.52
Information about health consequences	5	4.41
Information about emotional consequences	1	0.65
Action planning, implementation intentions	4	4
Report whether or not the behaviour was performed	2	1.74
Social reward	2	1.4
Habit formation	24	19.58
Social support (unspecified)	3	2.29

Note. N=17 patients. Data reported as average number. The average number of the single BCTs reported in this table, excludes the number of BCTs included in the "tailored to baseline questionnaire, integration of tailored BCTs".

Understanding and Actions upon Intervention Content

Participants reported that the intervention content increased their awareness about medication adherence and the risk and benefits of maintaining adherent to prescribed medications for long term, reinforced social support and habit formation, and reminded them to take medications as prescribed (table 4, Appendix 7).

Table 4. Participants' experiential feedback about the intervention content

Elements of the Intervention	Themes and quotes
Message content and tailoring<br><br>*Understanding and acceptability of the theoretical determinants*	Increase awareness of the importance to take medications as prescribed<br>"*So, that message came over quite clear, you know, that you must – you mustn't miss them. You must take them, you know. So, I thought it was quite good.*" patient 01001X<br>Increase awareness of the benefits to keep taking medications as prescribed, and potential risks when not taking medications<br>Q. *Can you remember a specific message*?<br>R. "*One message said how important it was to keep taking your pills to keep*

For peer review only

	you fit and healthy" patient 02028K<br>R. "*one [message] talked about the benefits of taking your medication regularly*" patient 02076E<br>R. "*If I'm honest, no. I've had a holiday in-between. No, I don't – I think probably, the one about – there was one about keeping – you're taking it to keep well, yeah, probably that one, that's the one that sticks in my mind*" patient 01051L<br>Remind to take medications<br>"*For me, the short message was ideal, you know? Who am I? and yes, and, "Have you taken your medication?"*" patients 01006W<br>"*I liked the idea of just a phone call to say, "Is that {name}?" "Yeah." "Take your tablet." Straightforward, you know*" patient 01001X<br>"*Oh, well, just the, you know, jogging the memory… Yeah, it's, you know, the telephone rings, somebody comes out with a message, are you so and so? Don't forget to take your tablets and so, you take them*" patient 01025G<br>"*I will say I liked it because it reminded me, like helped me to remember to take my tablet*" patient 01051L<br>"*I thought it was a good system to – it just reminded me to take my tablets*" patient 02028K<br>Social support<br>"*it was interesting to do it and to think someone's taking some notice of what I'm doing, trying to get rid of my high blood pressure*" patient 01024X<br>Habit formation<br>"*Well, the telephone call comes through at the same time every morning and so, you know you're going to take the pills.*" Patient 01025G<br>"*Well, I mean, yes, I mean, the phone call alone, without any message at all, would remind you*" patient 01031Q<br>"*It helped the routine, I mean, that was just that reminder of doing things ... by the end I'd developed a bit of a routine that I'm sitting there with the kind of phone ready for {time} o'clock, right, and then I would go and take my tablets*" patient 01057V

Note: Data from patients' (n=17) face-to-face interviews at the end of the one-month pilot intervention (T2).

Participants suggested that the intervention was acceptable and easy to use, and they provided their views about specific features of the intervention delivery mode and content. Specifically, the tailored schedule of the messages, the personalisation, and the variation of the content was found to be particularly appealing and were perceived to promote engagement with the intervention (Table 5, Appendix 6).

Table 5. Participants' experiential feedback about the intervention delivery mode.

Theme	Quote
Usability of the intervention, delivery mode, outbound and inbound calls	"*It was certainly easy to use. There's nothing complex about it really. I mean, if you've got any questions, you can just ring the numbers. I didn't have any questions, personally, but if you've got any, you can ring the number and it's quite easy and straightforward*" patient 01006W<br>"*It was always dead on time too, and if I – so, it didn't recognise it, they phoned back, sort of, about five minutes later and then it was alright*" patient 01024X<br>"*Well, it was very easy, just – yeah, very easy. I had clear numbers to call if there was a*

For peer review only - http://bmjopen.bmj.com/site/about/guidelines.xhtml

	problem, which I did, on a couple of occasions. No, it was very easy … I think having the choice of time is best… Yes, yeah, no, I think that is important, the choice of time, definitely" patient 02028K<br>"*It was easy and as long as it was at a set time, which it was, and there's – and if there's a second mobile phone, so if the first one misses, at least you can get hold of the person on the mobile if they're out and about*" patient 01025G

Note: Data from patients' (n=17) face-to-face interviews at the end of the one-month pilot intervention (T2).

Participants also recommended that some messages could be better received if delivered by either an IVR or SMS, depending on the timing and the content of the messages. For example, they recommended text messages at the time of medication taking with simple reminders of the behaviour to reinforce habit formation; whereas they recommended voice messages at a time of their preference with information about how to set up a routine with medication taking. They also suggested messages that target patients' INA beliefs and coping plans, to be delivered less frequently than reminders of the behaviour, as they were more personal and therefore easier to be perceived as invasive.

> "*So maybe a text message … 'cause you're waiting for the phone and you've got to do something, which, you know, I think is a good thing, in that you've got to do something and you have to respond. You can't just put your phone in your pocket and walk away, but at night time, I think it would probably be a bit intrusive*" patient 01062T, follow up interview.

Participants reported being satisfied with the overall experience with the intervention, the availability of the intervention 24/7, and their ability to call in and leave a message (see Appendix 6). They would also recommend the intervention to other people who take prescribed medications (see Appendix 7).

Discussion

Principal Findings

A highly tailored intervention has been developed to support adherence to medication prescribed for high blood pressure and co-morbidities. The intervention has been systematically developed based on guidance from MRC framework, PPI/E and is based on rigorous theory and evidence. It includes a combination of Behavioural Change Techniques (BCTs) tailored to patients' beliefs (e.g., beliefs about medications, self-efficacy, social norms, perceived regimen complexity) and prescription plan. The the pilot study suggested

For peer review only - http://bmjopen.bmj.com/site/about/guidelines.xhtml

that the intervention content increased awareness about the necessity to medication adherence, the risk and benefits of maintaining adherent to prescribed medications, reinforced social support and habit formation, and reminded patients to take medications as prescribed. To our knowledge, this is the first medication adherence intervention delivered using automated text and voice messages that has been developed and piloted within the UK primary care setting. The intervention has been proven to be acceptable and feasible, and it is currently evaluated in a randomised controlled trial.

Strengths and Limitations of this study

To date there are studies describing the systematic development of mHealth interventions, however none of them has provided a detailed description based on guidance from the MRC framework, and none has described the methods used to develop and pilot the implementation procedures within the primary care setting. Furthermore, this study has involved PPI/E in the intervention development, design and pilot, adapting a collaborative approach, which minimized the influence of the social desirability effect on the findings, and thus it provided a real-world perspective. As a consequence, the results of this intervention may be better informed and matched to patients' healthcare needs.

A strength of this study is the systematic approach to intervention development and piloting. The study used a rigorous theoretical approach to guide the development of the tailored intervention content. It also designed a flexible and scalable application to deliver messages to support adherence to different type, and complex prescribed medications. Another strength of this study is the utilisation of a mixed (e.g., qualitative, quantitative) and multi-perspective (patients, health care providers, PPI, stakeholders) methods of data collection. The data were combined complementarily into one comprehensive analysis, using a rigorous analytical approach.

Although this intervention is highly tailored, and participants can provide real time input and further tailor intervention content and delivery, a limitation of the intervention is the self-reported theoretical determinants of medication adherence. Future studies could enable the use of objectively measured theoretical determinants and objectively measured behaviour, using sensing technology. Another constrain of the text and voice messaging is its limited features to facilitate delivery of BCTs visually (e.g., picture of tablets, graphs to

For peer review only

show levels of adherence) [30], and future studies could usefully explore whether and how these features could influence intervention engagement and behaviour change.

Future Research

The one-month intervention has been extended to three months intervention and targets all adults (above 18 years old) to increase reach. It also includes another level of tailoring, where participants select between two BCTs to support maintenance of medication taking (e.g., habit formation or self-monitoring). The intervention is currently being evaluated in an RCT, which includes objective behavioural (medication event monitoring system) and clinical (systolic blood pressure, HbA1c) outcome measurements to inform the results about the efficacy of the intervention (see: https://doi.org/10.1186/ISRCTN10668149).

Funding statement: This paper presents independent research funded by the National Institute for Health Research (NIHR) under its School of Primary Care Research [grant number: RG86506] and the Research for Patient Benefit programme [grant number PB-PG-0215-36032]. The views expressed are those of the authors and not necessarily those of the NHS, the NIHR or the Department of Health. The funder had no role in study design, data collection, data analysis, data interpretation, the writing of the manuscript, and decision to submit the manuscript for publication.

Acknowledgment: This paper presents independent research funded by the National Institute for Health Research (NIHR) under its School of Primary Care Research [grant number: RG86506] and the Research for Patient Benefit programme [grant number PB-PG-0215-36032]. The views expressed are those of the authors and not necessarily those of the NHS, the NIHR or the Department of Health. The funder had no role in study design, data collection, data analysis, data interpretation, the writing of the manuscript, and decision to submit the manuscript for publication.

Author's contributions: AK conceived and designed the studies, developed the intervention, and led the intervention piloting, data collection, analyses, and wrote this manuscript. VH assisted in data collection. SE and JB provided IT expertise in developing the

For peer review only - http://bmjopen.bmj.com/site/about/guidelines.xhtml

digital platform. SS provided guidance and consultation to AK during all stages of the intervention development and piloting process.

Conflict of interest: the authors declare no conflict of interest.

Ethics approval and consent to participate: The Office for Research Ethics Committees of Northern Ireland (ORECNI; REC references: 16/NI/0150) and Essex East of England (REC Reference number 17/EE/0203), and the Health Research Authority, have reviewed and approved this project.

Data sharing. Qualitative and quantitative data are available upon request to AK.

References

1. NICE. *Hypertension in adults: diagnosis and management.* Available from: https://www.nice.org.uk/guidance/cg127
2. The Emerging Risk Factors Collaboration. *Association of Cardiometabolic Multimorbidity With Mortality.* Jama, 2015. **314**(1): p. 52-60.
3. Public Health England. *Tackling high blood pressure. From evidence into action*. 2014; Available from: https://www.gov.uk/government/publications/high-blood-pressure-action-plan.
4. Sackett D. *Compliance with therapeutic regimens*. 1976. The John hopkins University. Baltimore & London.
5. Mazzaglia G, Ambrosioni E, Alacqua M, Filippi A, Sessa E, Immordino V, et al., *Adherence to antihypertensive medications and cardiovascular morbidity among newly diagnosed hypertensive patients.* Circulation, 2009. **120**(16): p. 1598-605.
6. Chowdhury R, Khan H, Heydon E, Shroufi A, Fahimi S, Moore C, et al. *Adherence to cardiovascular therapy: a meta-analysis of prevalence and clinical consequences.* Eur Heart J, 2013. **34**(38): p. 2940-8.
7. Cassell A, Edwards D, Harshfield A, Rhodes K, Brimicombe J, Payne R, et al. The epidemiology of multimorbidity in primary care: a retrospective cohort study. Br J Gen Pract, 2018.

For peer review only

8. NCD Risk Factor Collaboration. *Worldwide trends in blood pressure from 1975 to 2015: a pooled analysis of 1479 population-based measurement studies with 19.1 million participants.* Lancet, 2017. **389**(10064): p. 37-55.

9. NICE. *Medicines Optimisation: The Safe and Effective Use of Medicines to Enable the Best Possible Outcomes*. 2015, National Institute for Health and Care Excellence (UK)

10. NICE. *Medicines Adherence: Involving Patients in Decisions About Prescribed Medicines and Supporting Adherence*. 2009, Royal College of General Practitioners (UK)

11. Tsoli S, Sutton S, Kassavou A. *Interactive voice response interventions targeting behaviour change: a systematic literature review with meta-analysis and meta-regression* BMJ Open 2017;0:e018974. doi:10.1136/bmjopen-2017-018974

12. Kassavou A, Sutton S. *Automated telecommunication interventions to promote adherence to cardio-metabolic medications: meta-analysis of effectiveness and meta-regression of behaviour change techniques.* Health Psychol Rev, 2017: p. 1-18. doi: 10.1080/17437199.2017.1365617.

13. Smith DH, O'Keefee-Rosetti M, Owen-Smith AA, Rand C, Tom J, Vupputuri S, et al. *Improving Adherence to Cardiovascular Therapies: An Economic Evaluation of a Randomized Pragmatic Trial.* Value Health, 2016. **19**(2): p. 176-84.

14. Hartmann-Boyce J, Lancaster T, Stead LF. *Print-based self-help interventions for smoking cessation.* Cochrane Database Syst Rev, 2014(6): p. Cd001118.

15. Yardley L, Morrison L, Bradbury K, Muller I. *The person-based approach to intervention development: application to digital health-related behavior change interventions.* J Med Internet Res, 2015. **17**(1): p. e30.

16. Mummah SA, King AC, Gardner CD, Sutton S. *Iterative development of Vegethon: a theory-based mobile app intervention to increase vegetable consumption.* Int J Behav Nutr Phys Act, 2016. **13**: p. 90.

17. Michie S, Atkins L, West R. *The Behaviour Change Wheel. The guide to developing interventions.* 2014, London

For peer review only - http://bmjopen.bmj.com/site/about/guidelines.xhtml

18. Craig P, Dieppe P, Macintyre S, Michie S, Nazareth I, Petticrew M, et al., Developing and evaluating complex interventions: the new Medical Research Council guidance. Bmj, 2008. 337: p. a1655.

19. Dijkstra A, De Vries H. *The development of computer-generated tailored interventions.* Patient Educ Couns, 1999. **36**(2): p. 193-203.

20. Kreuter MW, Farrell D, Olevitch L, Brennnan L. *Tailoring Health Messages. Customizing communication with computer technology*. Lawrence Erlbaum Assoc, 1999.

21. Barber N. *Should we consider non-compliance a medical error?* Qual Saf Health Care, 2002. **11**(1): p. 81-4.

22. Kassavou A, Sutton S. *Developing and pre-testing a tailored interactive voice response (IVR) intervention to support adherence to anti-hypertensive medications.* 2016, research protocol, The University of Cambridge.

23. Michie S, Richardson M, Johnston M, Abraham C, Francis J, Hardeman W, et al., *The behaviour change technique taxonomy (v1) of 93 hierarchically clustered techniques: building an international consensus for the reporting of behaviour change interventions.* Ann Behav Med, 2013. **46**(1): p. 81-95.

24. Farmer A, Hardeman W, Hughes D, Prevost AT, Kim Y, Craven A, et al. *An explanatory randomised controlled trial of a nurse-led, consultation-based intervention to support patients with adherence to taking glucose lowering medication for type 2 diabetes.* BMC Fam Pract, 2012. **13**: p. 30.

25. Kassavou A, Sutton S. *Reasons for non-adherence to cardiometabolic medications, and acceptability of an interactive voice response intervention in patients with hypertension and type 2 diabetes in primary care: a qualitative study.* BMJ Open, 2017. **7**(8): p. e015597.

26 Wilkinson S. *Qualitative data collection: interviews and focus groups*, in *Research methods for clinical and health psychology*, Yardley, L., Marks D, Editor. 2004. Sage Publications Ltd.

27. Alexander V, Thomas H, Cronin A, Fielding J, Moran-Ellis J. *Mixed methods* in *Researching Social Life*, N. Stoneman, Editor. 2016: London p. 119-138.

For peer review only

28. Moran-Ellis J. *Following a Thread – An Approach to Integrating Multi-method Data Set.'* Paper given at ESRC Research Methods Programme, Methods Festival Conference, Oxford, July 2004.

29. O' Cathain A, Murphy E, Nicholl J. Three techniques for integrating data in mixed methods studies. BMJ, 2010; 341:c4587.

30. Armitage L, Kassavou A, Sutton S. A systematic review of the literature with meta analysis and meta regression: the use of smartphone applicatons to suport medication adherence. Prospero protocol, 2018.

For peer review only

Appendix 1. Intervention development process, informed by the MRC Framework

Appendix 2. Intervention piloting process, informed by the MRC framework

Appendix 3: Map of the theoretical framework, determinants and BCTs to produce changes in adherence to medications.

For peer review only - http://bmjopen.bmj.com/site/about/guidelines.xhtml

Appendix 4: PPI/E messages

Appendix 5: Example of message tailoring.

Appendix 6. Participants' satisfaction with the intervention components

Appendix 7. Participants' perception of the intervention utility and future use

For peer review only

Appendix 1. Intervention development process, informed by the MRC Framework.

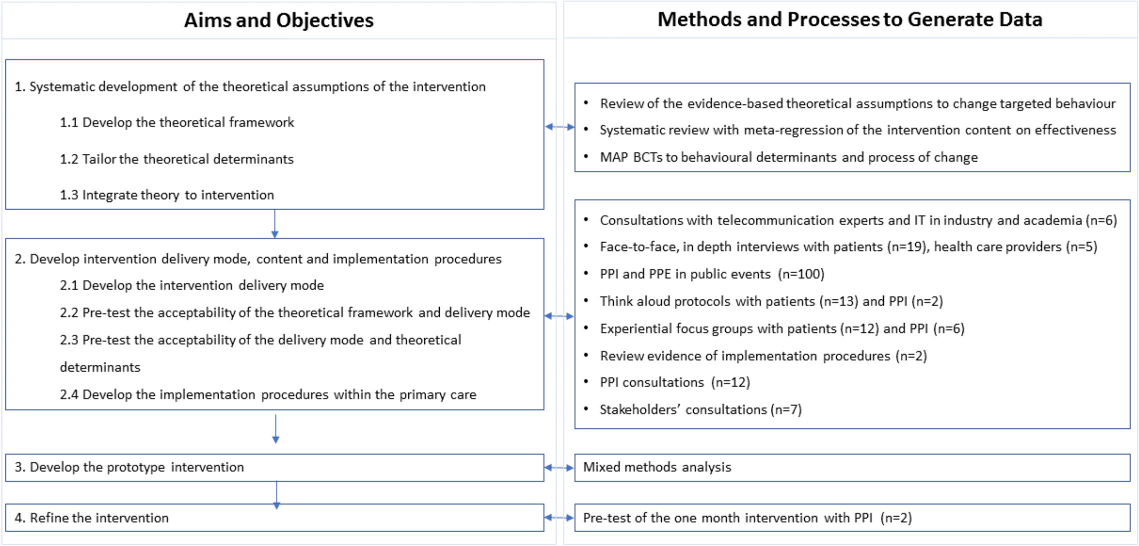

For peer review only

Appendix 2. Intervention piloting process, informed by the MRC framework.

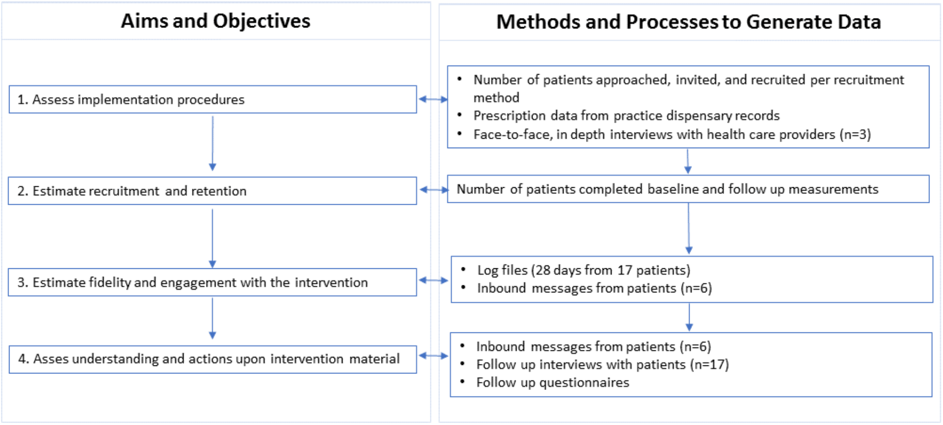

For peer review only

Appendix 3. Map of the BCTs to theoretical determinants and framework.

	Framework	Theoretical Determinant	BCTs
PRIMARY DETERMINANTS	INA	**Beliefs and knowledge**	*Re-attribution*
			Pros and cons
			Information about antecedents
			social comparison
			reduce negative emotions
			normative information
		**outcome expectations**	*Information about health consequences*
			information about emotional consequences
			Information about social and environmental consequences
			Pros and cons
			goal setting (behaviour)
			goal setting (outcome)
			self talk
			Social support (unspecified)
			Social support (practical)
			Socia support (emotional)
		**self efficacy**	*feedback on behaviour*
			reporting whether or not the behaviour was performed
			Problem solving
			action planning/ implementation intentions
			self monitoring of behaviour
			adding objects to the environment
			feedback on outcomes of behaviour
			biofeedback
			review behaviour goals
	NINA		*review outcome goals*
			feedback on emotional state
			monitoring of emotional consequences
			social reward
			Social support (unspecified)
			Social support (practical)
			Socia support (emotional)
			self talk
		**habit**	*Prompts/cues*
			habit formation
			action planning/ implementation intentions
			feedback on behaviour
			Social reward
SECONDARY DETERMINANTS		**social support**	*social comparison*
			social reward
			Social support (unspecified)
			Social support (practical)
			Socia support (emotional)
		**emotional/ affective state**	*Information about emotional consequences*
			Report emotional state
			Feedback on emotional state
			Monitoring of emotional consequences

Appendix 4. Examples of Patient and Public Involvement and Engagement (PPI/E) messages.

Scenario: Mark is a 49 year old car mechanic. He was diagnosed with high blood pressure by his GP 6 months ago even though he had no symptoms, was not overweight, did not smoke, ate healthily and had an active job. Initially, he took the medication exactly as his GP prescribed every day. However, after a few weeks he began to experience constipation and read on the leaflet supplied with the tablets that this was a side effect caused by the medication. Now, he only takes his medication every other day and the constipation has cleared up. However, his blood pressure is still too high.

Message	Event
Keep on the tablets prescribed to you by your Doctor. High blood pressure is more serious than constipation.	HOP
His action was foolish – he should have consulted his GP before changing the prescribed medication.	HOP
This scenario similar to mine. However I do take my meds without fail. I am motivated by my family history which leads me to believe I may be susceptible to heart disease which, of course, is thought to be related to blood pressure. I can understand why people might be concerned about side effects, so they need motivation.	HOP
I know that mechanics are frequently asked to work through lunch breaks etc. Please take some fruit and water to work with you to snack on throughout the day. This will help with your constipation, as you should be taking your pills every day. Good luck.	HOP
You need to talk to your GP in order for him to prescribe a laxative to help with the constipation. The need to take your BP medication is crucial in bringing your BP down.	HOP
BP tablets must be taken every day so consult your GP for medication for constipation.	HOP
It's stupid not to take your meds! If you're constipated just eat more fibre. It's not worth the risk.	HOP
Hey Mark. Are you aware of your blood pressure today? Have you taken your meds yet?	SF
Hi Mark. I sympathise a lot. I used to suffer from blood pressure and constipation, but nowadays I rarely suffer from either. I've had heart surgery but still cycle a lot. Don't give up on the meds. There are different tablets available: ask your GP. But also, use whole brand cereals regularly, even at bedtime!	SF
Hi Mark. You've sorted the constipation – keep going for the blood pressure!	SF
Mark, you should keep taking your medication even with the side effects, because it is better for you.	SF

Abbreviations: Hospital Open Event – HOP; Science Festival – SF; Focus Groups – FG

For peer review only

Appendix 5. Examples of tailoring messages.

Tailored theoretical determinants	BCT	Message file
↑ NINA and ↑ emotional state	Habit formation	Please do not forget to take your tablets. To achieve better control over high blood pressure, you will need to take them every day.
NINA / INA and ↓ social norms	Report whether or not the behaviour was performed, social reward, and information about health consequences	IVR: We are calling to see how you are doing with taking your tablets. Have you taken all your tablets as prescribed today?<br>P: yes<br>IVR: Well done! Taking your medications as prescribed is an easy way to keep blood pressure down.
↑ NINA	Habit formation	You are soon due to take your tablet. Please do not forget to do so.
NINA / INA and prescription plan	Habit formation and action planning	This is your message for your blood pressure tablets. One easy way to remember your tablets, is to take them with another daily activity, such as your morning cup of tea. If they are always done together, it will be harder to forget.
↑ Beliefs about medications, ↑ self-efficacy to take medications when no health benefits and ↑ emotional state	Habit formation and information about emotional consequences	Please keep taking your tablets as prescribed even if you are well and feeling healthy. High blood pressure is one of those things that unless you actually feel it you're not aware that it is a problem. Taking your medications as prescribed will support you to keep enjoying things or activities that are important to you.
NINA, ↓ beliefs about medications and ↓ emotional state	Habit formation and social support (unspecified)	Whatever the day may holds, please do not forget to take your tablets. To achieve better control over high blood pressure, you will need to take them every day.
↓ self-efficacy to cope with ↓ practical social support	Habit formation and social support (unspecified)	We are calling to see how you are doing with taking your tablets today. Please remember that you can always call the free telephone number if you require more support on how to take your tablets as prescribed. If you require more information about the health condition or the dosage of your medications, please contact your GP practice or pharmacist.
↑ social norms and ↑ self-efficacy to cope with ↓ practical social support	Habit formation and social support (unspecified)	You may find it helpful to ask people who are important to you to remind you to take your medications as prescribed and maintain your blood pressure under control. Please remember that you can always call the free telephone number if you require more support on how to take your tablets as prescribed.

Note: ↑indicate higher score from the cut-off point, ↓ indicate lower score from cut-off point.

For peer review only

Appendix 6. Participants' satisfaction with the intervention components

	Mean (sd)
The experience with the intervention	4 (0.7)
The voice delivering the intervention messages	3.7 (1.1)
The content of the intervention messages	3.8 (0.8)
Being able to call the intervention	4 (0.7)
The availability of the intervention 24/7	4.2 (0.8)
The response you received from the intervention	3.4 (0.7)
The response you received to your particular queries/questions	3.4 (0.7)

Note: Likert scale, 1 strongly agree to 5 strongly disagree. Data from n=17 participants.

For peer review only

Appendix 7. Participants' perception of the intervention utility and future use

	Mean (sd)
The intervention messages helped me to take my tablets as prescribed	3.2 (1.3)
The intervention reminded me to take my medications as prescribed	3.5 (1.3)
The intervention was easy to use	4.3 (0.7)
The intervention addressed my personal needs	3.3 (1.1)
It would have been useful, if the intervention was part of the regular care	3.7 (0.9)
I would recommend the intervention to other people who take medications for long-term health conditions	4.2 (0.8)

Note: Likert scale, 1 strongly agree to 5 strongly disagree. Data from n=17 participants.

For peer review only

BMJ Open

Development and piloting of a highly tailored text and voice messaging intervention to support adherence to antihypertensive medications within the primary care setting

Journal:	*BMJ Open*
Manuscript ID	bmjopen-2018-024121.R1
Article Type:	Research
Date Submitted by the Author:	24-Jul-2018
Complete List of Authors:	Kassavou, Aikaterini; University of Cambridge, Public Health and Primary Care ; University of Cambridge,<br>Houghton , Vikki<br>Edwards, Simon<br>Brimicombe, James; University of Cambridge, Primary Care Unit, Department of Public Health and Primary Care<br>Sutton, Stephen; University of Cambridge,
**Primary Subject Heading**:	Public health
Secondary Subject Heading:	Cardiovascular medicine, Health services research, Public health
Keywords:	medication adherence, Hypertension < CARDIOLOGY, interactive voice response, text messages, pilot intervention

SCHOLARONE™
Manuscripts

INTERVENTION DEVELOPMENT AND PILOTING

Development and piloting of a highly tailored text and voice messaging intervention to support adherence to antihypertensive medications within the primary care setting

Aikaterini Kassavou, Vikki Houghton, Simon Edwards, James Brimicombe, and Stephen Sutton

Address: University of Cambridge, School of Clinical Medicine, Department of Public Health and Primary Care, Forvie Site, Robinson Way, Cambridge, CB2 0SR

Corresponding author: Aikaterini Kassavou

Email: kk532@medschl.cam.ac.uk

Address: University of Cambridge, School of Clinical Medicine, Department of Public Health and Primary Care, Forvie Site, Robinson Way, Cambridge, CB2 0SR.

For peer review only

Key words: medication adherence, hypertension, interactive voice response, text messages.

Word count: 4920

Abstract

Objectives: This paper describes the systematic development and piloting of a highly tailored text and voice message intervention to increase adherence to medication within primary care.

Methods: following the MRC guidance this paper describes: a) the systematic development of the theoretical framework, based on review of theories and meta-analyses of effectiveness; b) the systematic development of the delivery mode, intervention content and implementation procedures, based on consultations, face-to-face interviews, think-aloud protocols, focus groups, systematic reviews, PPI/E input, intervention pre-test, with c) the piloting of the intervention, based on a one-month intervention; and follow up assessment including interviews and questionnaires. The mixed methods analysis combined findings from the parallel studies complementarily.

Results: intervention development suggested the target behaviour of the intervention should be the tablets taken at a regular time of the day. It recommended that patients could be more receptive to intervention content when they initiate medication taking or they change prescription plan; and more emphasis is needed to patients' consent process. Intervention piloting suggested high intervention engagement with, and fidelity of, the intervention content; which included a combination of BCTs, and was highly tailored to patients' beliefs, and prescription plan. Patients reported that the intervention content increased awareness about the necessity to take and maintain adherent to medication, reinforced social support and habit formation, and reminded them to take medication as prescribed.

Conclusion: tailored automated text and voice message interventions are feasible ways to increase medication adherence, as an adjunct to primary care.

Keywords: adherence, anti-hypertensive medication, text messaging, interactive voice response.

For peer review only - http://bmjopen.bmj.com/site/about/guidelines.xhtml

Strengths and limitations of this study

- This is the first medication adherence intervention for patients with hypertension and co-morbidities that has been developed and piloted within the UK primary care setting.
- The study used rigorous methodology to collect and analyse data, from multiple perspectives.
- This study involved and engaged Patients and Public in all stages of the intervention development and piloting.
- Future studies should include real-time and objective measures of the intervention determinants and outcomes.

For peer review only

Introduction

Hypertension or high blood pressure (HBP), affects approximately one in four people in England [1], and often accompanies other conditions, including type 2 diabetes mellitus (T2DM), coronary heart disease and stroke [2]. These conditions are major risk factors for disability and premature death [2], and medication adherence can significantly lower these risks [1]. For example, in England over ten years, an estimated 7,000 quality-adjusted life years (QALYs) could be saved and £120m not spent on related health and social care, if people had better adherence to anti-hypertensive medications [3].

Adherence is defined as taking at least 80% of the prescribed tablets, and/or having medications dispensed for at least 95% days of a prescribed period [4]. However, substantial proportions of patients do not take their medication as prescribed [5]. A recent meta-analysis showed that 40% of people do not adhere adequately to cardiovascular medication and the percentage was similar (41%) for non-adherence to anti-hypertensive medications [6]. Taking into consideration the growing prevalence of HBP, co-morbidities and the ageing population [7], especially in lower and middle-income countries [8], it is likely that there will be an increase in demand for health care resources to support medication adherence. The National Institute of Health and Care Excellence (NICE) recommends the development of novel and cost-effective interventions to aid patients' decision making about taking medicines, as an adjunct to health care providers' consultations [9, 10].

Tailored mHealth interventions, such as text (SMS) and voice (IVR) messaging intervention, is one way to support patients' adherence between their consultations. Review evidence found that such interventions can effectively support adherence to different types of medication, including antihypertensive tablets, when compared to usual care only [11, 12], and can potentially be cost-effective if applied to a large number of people [13]. For example, a recent study suggested that such interventions could result in a 2.3% increase in QALYs and overall savings of 21,120 Australian dollars during a patient's lifetime [14].

For peer review only - http://bmjopen.bmj.com/site/about/guidelines.xhtml

For peer review only

However, no such intervention has been developed and piloted in the UK primary care setting.

The aim of this paper is to describe the development and piloting of a highly tailored text and voice messaging intervention to support adherence to antihypertensive tablets. Although several guidelines have been proposed to the development of mHealth interventions [15-17], this paper has been informed by the Medical Research Council (MRC) Framework [18] as it leads not only the process to develop the intervention content and delivery mode, but also the implementation procedures within the primary care National Health Service (NHS), which is an important process in developing and piloting novel interventions.

Methods

Review of theory and evidence, interviews, focus groups, consultations and Patients and Public (PPI) involvement and Engagement (PPE) informed the intervention development process (see Appendix 1); and a one-month pre-post intervention study, with follow up assessment informed the piloting of the intervention (see Appendix 2).

Intervention Development

Step 1. Systematic Development of the Tailored Intervention.

The systematic development of the tailored intervention was informed by the guidance provided by Dijkstra and De Vries [19] and Kreuter and colleagues [20] on the process of developing computer telephony interventions. This process included the development of the theoretical framework, tailoring, and integration.

Develop the theoretical framework. The theoretical framework that distinguishes between intentional non-adherence (INA) and non-intentional non-adherence (NINA) guided the development of this intervention [21]. To address INA and NINA, we identified the empirical evidence of the proximal and modifiable determinants of adherence to medication [22] and mapped them into INA and NINA framework. Following that, we used the Taxonomy of the Behaviour Change Techniques (BCTs) [23] to identify the techniques

For peer review only - http://bmjopen.bmj.com/site/about/guidelines.xhtml

that impact on INA and NINA determinants (see Appendix 3). Our decision on the BCTs for this intervention was informed by findings from two systematic literature reviews [11, 12] and previous empirical evidence [24].

Tailor the theoretical determinants. To develop a highly tailored intervention we produced a tailoring matrix, which consists of a theory-based questionnaire, an algorithm of decision rules, the schedule and the message file. The theory-based questionnaire includes items measuring each participant's values of the theoretical determinants. The algorithm includes decision rules (i.e., cut-off points), which facilitates the decisions on what message to prioritize and indicates a default feedback. The tailoring process of the intervention messages is informed by multiple sources, which can be grouped in three 'clusters': the first cluster of tailoring is based on participant's unique characteristics (e.g., name) and is informed by records from the primary care practices; it aims to gain person's attention to the intervention by making messages personally relevant. At the second cluster of tailoring we use data from participant's responses at the tailoring questionnaire and their prescription plan. This cluster includes two sub-levels of tailoring: tailoring based on the degree of (a) INA and NINA, and (b) each of the modifiable INA and NINA theoretical determinants. At the third cluster, we tailor the intervention to participant's responses and feedback during the intervention. Participants are able to further tailor the intervention content by requesting a higher or lower degree of information for a particular determinant or by requesting feedback on a different determinant. Intervention tailoring at this cluster takes into consideration participant's navigation options until the request. The schedule includes the frequency of the messages for the duration of the intervention, which participants can increase, reduce or stop. The message file included the tailored BCTs.

Integrate theory to intervention. The frequency, combination and sequence of the BCTs were integrated into one intervention. The decision on the above elements was based on the phase participants are in (e.g., uptake or maintain), having as the starting point the collection of the medication from the dispensary.

Step 2. Development of the Intervention Delivery Mode, Content, and Implementation Procedures.

For peer review only - http://bmjopen.bmj.com/site/about/guidelines.xhtml

INTERVENTION DEVELOPMENT AND PILOTING

This step of the intervention development aimed to convert the theoretical underpinnings into features of the delivery mode, intervention content, and implementation procedures, and it involved: the development of the delivery mode, the pre-test of the theoretical framework and the delivery mode, the pre-test of the delivery mode and the theoretical determinant, and the development of the implementation procedures within primary care.

Develop the intervention delivery mode. To translate the theoretical underpinning into features of the intervention delivery mode, consultations with six experts from the industry and academia were conducted. Meetings with telecommunication and IT experts in the University of Cambridge informed the decisions about the development of the IVR application. The discussions informed the development of features of the IVR applications, which involved the navigation options, the degree of interactivity, the voice delivering the messages, and the prompts to facilitate participants' navigation and dialogue. The discussions for the development of the SMS application involved the necessary features to accommodate messages on complex prescription plans. A custom written database application contained the functionality to create output for both delivery modes, and data were transferred at regular intervals to both platforms (IVR and SMS) using secure data hosting protocols. The digital platform could also facilitate participants' feedback during the intervention or at a different time (i.e., by triggering an inbound call). Voice recognition facilitated interaction and data collection. The digital platform enabled flexible schedule of messages delivery; for example, messages could be delivered at times tailored to participant's prescription plan (e.g., 1-4 messages per day).

Pre-test the acceptability of the theoretical framework and delivery mode. One interview study and Patients and Public Involvement (PPI) and Engagement (PPE) informed the acceptability of the theoretical framework and delivery mode.

Face-to-face, in depth interviews were conducted with patients and health care providers to assess the acceptability of the delivery mode, the theoretical framework, and generate relevant content. Patients were randomly identified and selected by the practice manager from practice databases. Primary care practices within the Cambridgeshire and Peterborough Clinical Commissioning Group (CCG) were eligible for inclusion, and five primary care practices from different areas of deprivation (based on Index of Multiple Deprivation; IMD) took part in the study. Patients were eligible if they: (a) had a diagnosis of

For peer review only - http://bmjopen.bmj.com/site/about/guidelines.xhtml

HBP, T2DM or both health conditions; (b) had been prescribed at least one anti-hypertensive and/or glucose lowering medication as confirmed by practice records for at least three months before recruitment; (c) had poorly controlled blood pressure or glucose levels as indicated by practice records or had gaps in collecting repeat prescriptions; and (d) were aged 40 years or older. Patients were excluded when they: (a) had hearing or speaking impairment, (b) had a diagnosis of dementia, aphasia or other cognitive difficulties that could affect the study participation, (c) had a recent severe life-threatening event, (d) had difficulty in speaking or understanding English, or (e) participated in another study. A practice GP double screened the list of selected patients against the inclusion and exclusion criteria to confirm eligibility. Eligible patients were invited by post or telephone calls following up a postage invitation. Health care providers (i.e., nurses and health care assistants) who had experience with advising patients about their medications were eligible to participate and interviewed in the practices. Nineteen patients and five health care providers were interviewed face-to-face. Both patients and health care providers' interview data informed inductively the content of the theoretical determinants and the BCTs, and provided their views on the acceptability of the automated intervention within the primary care setting.

Patients and Public Involvement (PPI) and Engagement (PPE) was conducted to assess acceptability of the delivery mode and the theoretical framework, and inform the intervention content. PPI/E members were recruited opportunistically using emails or adverts. In total 100 PPI/E members took part in three events: one meeting with a diabetes patients' group (n=20), one open event at Addenbrookes' Hospital (n=30), and one open event at the Cambridge Science Festival (n=50). During the three PPI/E events, members were asked their views and recommendations on the delivery mode, and to write messages tailored to INA and NINA case studies. Data generated from PPI/E informed the content of the message file (for example, see Appendix 4). Overall, PPI/E members found more acceptable NINA case studies and seemed to generate more messages for NINA rather than INA case studies.

Pre-test feasibility of intervention delivery mode and theoretical determinants. Four studies informed the acceptability of the delivery mode and the theoretical determinants. These included: think aloud interviews, experiential focus groups, and PPI.

For peer review only - http://bmjopen.bmj.com/site/about/guidelines.xhtml

INTERVENTION DEVELOPMENT AND PILOTING

Face-to-face, in depth interviews were conducted with 13 patients. Think aloud protocol was used to assess reliability of the mechanisms of change (i.e., link between the theoretical determinants and the BCTs), and usability of the delivery mode. All interviews were conducted at participants' places. Field notes and think aloud data informed the analysis, and results refined the tailoring of the theoretical determinants [25].

Face-to-face meetings were conducted with two PPI members recruited using emails from the list of the National Institute of Health Research (NIHR) Cambridge BRC PPI/E Panel and Communications office. Think aloud protocol was used to assess the reliability and sequence of the items to form the measurement of the theoretical determinants (i.e., theory-based questionnaire). Both interviews were conducted at the University. Field notes and think aloud data informed the analysis, and results refined the reliability of the tailoring questionnaire and informed the decision rules of the tailoring algorithm.

Experiential focus groups aimed to obtain patients' views of, and refine, the tailored intervention content (e.g., inclusion of medication name, dose and timings; 'please do not forget to take 1 tablet ramipril 10 mg at 9am') and delivery mode within a social interaction context [26]. Eligibility criteria and recruitment procedure were similar to patients' face-to-face interviews, reported above. Four focus groups took place in primary care practices (n=2) or community centres (n=2). During the focus groups patients (n=12) received examples of SMS and IVR messages on their mobile phones and were asked to provide experiential feedback. Patients were asked their views and recommendations about the combination of the delivery modes and the intervention content, using a think-aloud protocol.

One experiential focus group with six PPI members aimed to obtain views and recommendations on the intervention content and delivery mode. PPI were recruited using emails from the NIHR Cambridge BRC PPI/E office. PPI members were eligible to take part if they were prescribed medications for a long-term health condition and were over 40 years old.

The data generated from the experiential focus groups informed the content of the message file and the process to map the intervention messages onto the theoretical determinants.

For peer review only

Develop implementation procedures within primary care. The intervention implementation procedures were informed by systematic review evidence, PPI/E, and stakeholders' consultations.

Information about the recruitment setting and patients' characteristics at baseline, as well as uptake and retention rates were extracted from the two systematic reviews of trials on medication adherence delivered by SMS and/or IVR messages [11, 12]. Due to missing data about the characteristics of the targeted population, the recruitment setting, recruitment methods and material, we synthesised the findings narratively. Results informed the decisions regarding the recruitment setting and methods, as well as the targeted behaviour and population of the intervention.

Twelve PPI members provided feedback on the recruitment material (e.g., patients' information sheets, posters, leaflets, flyers, and invitation letters) using emails or face-to-face meetings. Their comments informed the content of the recruitment material and the description of the recruitment, informed consent, and intervention procedures in lay language.

Stakeholders' consultations were conducted with health care providers, commissioners, and patients to inform the study design and the implementation procedures within the primary care setting (e.g., recruitment, informed consent, data collection). Practitioners with experience of advising patients for medications; commissioners for medicine optimisation, prescribing or cardiovascular conditions; and patients with either HBP or both HPB and T2DM were included. Commissioners were recruited informally by email through personal networks. Patients and health care providers (i.e., practice nurses, health care assistants) were recruited through the primary care practices. Those who consented to take part were invited to the consultations. Consultation modes were flexible to maximise participation (n=3 face-to-face, n= 4 email). Stakeholders (N=7) were given a description of the proposed intervention and asked their views and recommendations. Health care providers (n=4) were consulted about the time to initiate recruitment, and the recruitment methods and procedures. Commissioners (n=1) were consulted about how best to track resource use and costs and the evidence needed to inform whether or not to commission such an intervention. Patients (n= 2) were consulted about the delivery mode and the theoretical framework. All stakeholders were asked for their recommendations

For peer review only - http://bmjopen.bmj.com/site/about/guidelines.xhtml

about the implementation procedures. The data generated from stakeholders' consultations informed the study and intervention implementation procedures.

Step 3. Develop the Prototype Intervention.

A prototype intervention was developed by multiple and iterative syntheses of the data obtained during the intervention development [27]. The intervention is highly tailored to deliver very brief (≥1 minute), theory-based messages at participant's preferred time, telephone and frequency to receive the calls. The pilot intervention consisted of 29 messages, delivered daily for a duration of one prescription-based month. Two messages were delivered at the first day of the intervention, with feedback tailored to each participant's responses at the baseline questionnaire and prescription plan, and one message per day during the following 27 days of the intervention.

Step 4. Refine the Intervention.

Two PPI members, recruited by the NIHR Cambridge BRC PPI/E office, pre-tested the prototype intervention for one prescription-based month. PPI provided their experiential feedback about the intervention usability and mechanisms of change during four, weekly telephone-based interviews with a researcher, and completed the follow up questionnaire. The data informed the decision rules of the tailoring algorithm and refined the frequency (i.e., message schedule), combination and sequence of the BCTs (i.e., integration of theory into the intervention), before piloting the prototype intervention within primary care.

Intervention Piloting

A one-month pilot intervention was conducted to assess (a) implementation procedures, (b) uptake, retention, fidelity, and engagement with the intervention, (c) participants' views, understanding, and actions upon the intervention content; and obtain (d) recommendations for improvement.

For peer review only - http://bmjopen.bmj.com/site/about/guidelines.xhtml

INTERVENTION DEVELOPMENT AND PILOTING

Patients with HBP were recruited by primary care practices within the Cambridgeshire and Peterborough CCG, and health care providers who consulted patients for taking medications. Patients were selected and recruited using the same recruitment procedure described in the intervention development studies but included two additional recruitment strategies: (a) face-to-face recruitment by health care providers, and (b) leaflets and posters in the practice waiting rooms. Health care providers received training by a researcher on the face-to-face recruitment procedures. Patients completed measures at baseline (T1) and at the end of the intervention (T2).

Patients completed the tailoring questionnaire (T1, T2), their preferred primary and secondary telephone number to receive the intervention messages, and the time intervals of repeated calls per telephone number (T1), two items measuring adherence (T1, T2), 16 items measuring their experience and satisfaction with elements of the intervention (T2), and face-to-face interviews with a member of the research team (T2). Medication collection data for the duration of three months before T1, and at T2, were obtained from practice dispensary records. Telephone log files assessed uptake, retention, fidelity, and engagement with the intervention. Participants' inbound calls assessed intervention engagement and impact. Face-to-face interviews (T2) assessed users' understanding and actions upon the intervention and obtained recommendations for improvement. Face-to-face interviews with health care providers (n=3) assessed implementation procedures and obtained recommendations for improvement.

Patients and Public Involvement

Patients and Public Involvement (PPI) and Engagement (PPE) was conducted to inform intervention development and piloting (see sections above).

Analysis

Data generated from the intervention development and pilot study were integrated into one mixed methods analysis [27], using the technique called "following a thread" [28, 29]. The theoretical underpinnings of the intervention were explored across different methods

For peer review only - http://bmjopen.bmj.com/site/about/guidelines.xhtml
For peer review only

of data collections, data generated from each method informed each intervention component (i.e., intervention content and implementation procedures) iteratively, and questions or contradictory findings generated were followed across the other methods until saturation was achieved.

Results

A summary of the outcomes of the mixed methods analysis are reported in table 1, and explained in more details at this section

Table 1. Outcomes and results from intervention development and piloting.

**Intervention development**	**Intervention piloting**
• Theory based questionnaire<br>• Tailoring algorithm<br>• Message file<br>• Message schedule<br>• Delivery mode<br>• Targeted behaviour and participants' characteristics<br>• Implementation procedures within the primary care	• Recruitment and retention rates<br>• Fidelity and engagement with the intervention<br>• Understanding and actions upon intervention material

Theory Based Questionnaire

Participants' feedback on the questionnaire suggested that self-reported measures of non-adherence have confounded effect on measures of psychological constructs of adherence, and thus an objective measure of adherence was recommended. For example, Medication Adherence Report Scale items were perceived to measure INA /NINA constructs, rather than taking or not taking medications.

Tailoring

Participants' input suggested amendments to the tailoring algorithm, to increase the sensitivity of the algorithm in selecting INA participants. For example, the cut-off points of the algorithm were amended to facilitate the identification of INA and NINA. Moreover, participants' input suggested that the theoretical determinants could be the same for INA and NINA but they might differ in terms of the value of tailoring, thus we adjusted the

For peer review only

theoretical model and cut-off points, so that each of the theoretical determinant could appear to both INA and NINA participants (for example, see Appendix 5, for examples of tailoring BCTs).

Message File

Participants suggested content that informed the intervention BCTs, so that we included more BCTs, such as information about social and environmental consequences.

> "*Do you, sort of, remind them of the costs and things? Is that in with medication and things, if they're missing it, or every time they lose blood pressure medication or throw it in the bin, what is the cost?* " Practitioner consultation

Schedule

The one-month intervention schedule included daily messages and participants could change the frequency or stop them. Patients at follow up interviews, recommended an intervention of longer duration with messages to gradually decrease in frequency and include advice and support for self-monitoring medication taking.

Targeted Behaviour and Participants' Characteristics

Patients reported that it was easier for them to report on the pills taken per day, rather than the specific type of tablets (e.g., felodipine, statins). However, they reported that this information could be useful for patients who initiate medication taking, and need more education about their prescribed regimens. Patients reported that the timing of medication-taking or the routine associated with the timing (e.g., lunch), rather than the type of medications to take, was an easier reminder of behaviour. That was particularly important for those patients taking medication for longer and for more than one health conditions.

> Q: "*How many tablets do you take per day?*" R: "*Oh, about 17*". Q: "*How many different types of medication do you take per days?*" R: "*Yeah. That takes more counting and a bit more thinking ... I suppose, as well, if you have so many, you just get used to the times…Morning, lunch, evening, night time*" patient

Patients who took part in the intervention development studies and the pilot study were recruited from different areas of IMD, were older adults, had either HBP or co-morbidities

For peer review only - http://bmjopen.bmj.com/site/about/guidelines.xhtml

(see appendix 6, for participants' demographic characteristics), and self-reported being prescribed complex medication regimens (number of tablets per day: mean=6, sd=4.6).

Implementation Procedures within Primary Care

Health care providers that conducted the recruitment procedures reported challenges with recruiting non-adherent patients and recommended recruitment strategies delivered at times when patients might be more receptive to uptake such an intervention. In accordance to the systematic review evidence, it was recommended that patients would be more receptive to uptake the intervention when they initiate medication taking or when there is a change to their current prescription plan (e.g., this could be either due to a health event and/or due change to the pharmaceutical product). Otherwise, patients could be reluctant to provide consent and uptake the intervention (table 2).

Table 2. Health care providers' experiential feedback

Theme	Quote
Views about recruiting non-adherent patients	*"[patients who do not adhere to medication] they're just not coming in for their monitoring … a lot of the names on the list [of eligible participants] were people that weren't coming in anyway and that was the biggest problem"* practice nurse
Views about barriers to recruit for a digital intervention	*"cause they thought it was them, they had bad blood pressure or something … there was a question about what other medication do you take as well, and they're like, 'Why do we have to answer that if it's only about the blood pressure one?' … but he felt like they were invading his space, sort of thing, by putting down all the medications, you can kind of guess what they're for, and that's what he didn't really want to"* practice nurse
Views and recommendations about recruitment methods	Annual reviews: *"annual review is a bit more time … when its annual reviews because we're actually talking about their tablets"* practice nurse recommendation about recruitment procedures<br>Practice website: *"but you can put a facility on the online forum. A lot of people access our website, so there are quite a lot of other places that people might look maybe more than a poster"* practice nurse<br>Medication prescriptions: *"but every time you get a repeat prescription list, you can write something on it"* practice nurse

Note. N=8 health care providers: nurses and health care assistants. Themes have been coded at interviews with health care providers at development (n=5) and piloting of the intervention (n=3). Quotes reported in table are from experiential interviews with health care providers (T2).

For peer review only - http://bmjopen.bmj.com/site/about/guidelines.xhtml

Health care providers recommended that recruitment methods could be integrated to medication annual reviews or to patients' prescriptions. They also recommended that non-adherent patients, who do not attend the practice for their medication reviews, either because they do not appreciate the need of these or they are sceptical about the recommended treatment, could be more receptive to the intervention if invited by other methods, such as text messages, newsletters, or multimedia (table 2).

However, health care providers also revealed that time constrains prevented them from explaining the implementation procedures to eligible patients during primary care consultations. In line with data from the PPI consultations and patients' interviews, health care providers suggested that there is a lot of information in the recruitment material and suggested that more emphasis is needed to patient's consent process. It was suggested that patients should be made aware about what information will be collected by their practice records, and how these will be utilised from the intervention. To facilitate patients' understanding and standardise the implementation procedures, including the informed consent, we developed videos aligned to written material, and integrated these into a brief face-to-face recruitment appointment.

To reinforce the uptake and integration of the intervention into current NHS, commissioners, patients, and health care providers reported that intervention needs to be of low or no cost. *"Would need to be very low cost or free to use"* commissioner consultation.

Intervention Uptake and Retention

Twenty patients were recruited. Eighteen provided written informed consent and registered into the study, of whom 17 completed the one-month intervention: one patient was excluded by the research team before registration because he/she met one of the exclusion criteria, one patient withdrew before registration to the intervention, and the other participant dropped out during the intervention. All 17 patients completed the measures at baseline and at the end of the intervention. All primary care practices provided completed baseline (e.g., refill prescription data three months before the start of the intervention) and follow up data, for all participants.

Intervention Fidelity and Engagement

For peer review only - http://bmjopen.bmj.com/site/about/guidelines.xhtml

INTERVENTION DEVELOPMENT AND PILOTING

During the one-month intervention an average of 29 messages were scheduled, of which 22.52 were received by patients. On average, 37 calls were made, of which 2.8 failed to be made due to technical issues and 13 calls were made but failed to go through because they were not picked up by the participants. On average, three calls were repeated and received by participants. Ten out of the 17 participants had chosen the calls to be repeated if not answered, and five out of the 17 participants had provided a secondary number for the calls to be repeated, if there was no answered at the primary number. Overall, patients were engaged with the intervention and made inbound calls to change the schedule of the messages or report about intervention content. In summary, there was a good fidelity of the intervention BCTs, with the majority of the BCTs having high fidelity scores (table 3).

Table 3. Fidelity and engagement, intervention content

Messages, including one or more BCTs	*Scheduled*	*Received*
Tailored to baseline questionnaire, integration of tailored BCTs	1	0.6
Personalised	29	22.52
Information about health consequences	5	4.41
Information about emotional consequences	1	0.65
Action planning, implementation intentions	4	4
Report whether or not the behaviour was performed	2	1.74
Social reward	2	1.4
Habit formation	24	19.58
Social support (unspecified)	3	2.29

Note. N=17 patients. Data reported as average number. The average number of the single BCTs reported in this table, excludes the number of BCTs included in the "tailored to baseline questionnaire, integration of tailored BCTs".

Understanding and Actions upon Intervention Content

Participants reported that the intervention content increased their awareness about medication adherence and the risk and benefits of maintaining adherent to prescribed medications for long term, reinforced social support and habit formation, and reminded them to take medications as prescribed (table 4, Appendix 7).

Table 4. Participants' experiential feedback about the intervention content

Elements of the Intervention	Themes and quotes
Message content and tailoring	Increase awareness of the importance to take medications as prescribed<br>*"So, that message came over quite clear, you know, that you must – you mustn't miss them. You must take them, you know. So, I thought it was quite*

For peer review only

Understanding and acceptability of the theoretical determinants	*good*." patient 01001X<br>Increase awareness of the benefits to keep taking medications as prescribed, and potential risks when not taking medications<br>Q. *Can you remember a specific message*?<br>R. "*One message said how important it was to keep taking your pills to keep you fit and healthy*" patient 02028K<br>R. "*one [message] talked about the benefits of taking your medication regularly*" patient 02076E<br>R. "*If I'm honest, no. I've had a holiday in-between. No, I don't – I think probably, the one about – there was one about keeping – you're taking it to keep well, yeah, probably that one, that's the one that sticks in my mind*" patient 01051L<br>Remind to take medications<br>"*For me, the short message was ideal, you know? Who am I? and yes, and, "Have you taken your medication?"* patients 01006W<br>"*I liked the idea of just a phone call to say, "Is that {name}?" "Yeah." "Take your tablet." Straightforward, you know*" patient 01001X<br>"*Oh, well, just the, you know, jogging the memory… Yeah, it's, you know, the telephone rings, somebody comes out with a message, are you so and so? Don't forget to take your tablets and so, you take them*" patient 01025G<br>"*I will say I liked it because it reminded me, like helped me to remember to take my tablet*" patient 01051L<br>"*I thought it was a good system to – it just reminded me to take my tablets*" patient 02028K<br>Social support<br>"*it was interesting to do it and to think someone's taking some notice of what I'm doing, trying to get rid of my high blood pressure*" patient 01024X<br>Habit formation<br>"*Well, the telephone call comes through at the same time every morning and so, you know you're going to take the pills*." Patient 01025G<br>"*Well, I mean, yes, I mean, the phone call alone, without any message at all, would remind you*" patient 01031Q<br>"*It helped the routine, I mean, that was just that reminder of doing things ... by the end I'd developed a bit of a routine that I'm sitting there with the kind of phone ready for {time} o'clock, right, and then I would go and take my tablets*" patient 01057V

Note: Data from patients' (n=17) face-to-face interviews at the end of the one-month pilot intervention (T2).

Participants suggested that the intervention was acceptable and easy to use, and they provided their views about specific features of the intervention delivery mode and content. Specifically, the tailored schedule of the messages, the personalisation, and the variation of the content was found to be particularly appealing and were perceived to promote engagement with the intervention (Table 5, Appendix 7).

Table 5. Participants' experiential feedback about the intervention delivery mode.

Theme	Quote
Usability of the intervention,	"*It was certainly easy to use. There's nothing complex about it really. I mean, if you've got any questions, you can just ring the numbers. I didn't have any questions, personally, but if you've got any, you can ring the number and it's quite easy and straightforward*" patient

delivery mode, outbound and inbound calls	01006W<br>*"It was always dead on time too, and if I – so, it didn't recognise it, they phoned back, sort of, about five minutes later and then it was alright"* patient 01024X<br>*"Well, it was very easy, just – yeah, very easy. I had clear numbers to call if there was a problem, which I did, on a couple of occasions. No, it was very easy … I think having the choice of time is best… Yes, yeah, no, I think that is important, the choice of time, definitely"* patient 02028K<br>*"It was easy and as long as it was at a set time, which it was, and there's – and if there's a second mobile phone, so if the first one misses, at least you can get hold of the person on the mobile if they're out and about"* patient 01025G

Note: Data from patients' (n=17) face-to-face interviews at the end of the one-month pilot intervention (T2).

Participants also recommended that some messages could be better received if delivered by either IVR or SMS, depending on the timing and the content of the messages. For example, they recommended text messages at the time of medication taking with simple reminders of the behaviour to reinforce habit formation; whereas they recommended voice messages at a time of their preference with information about how to set up a routine with medication taking. They also suggested messages that target patients' INA beliefs and coping plans, to be delivered less frequently than reminders of the behaviour, as they were more personal and therefore easier to be perceived as invasive.

> *"So maybe a text message … 'cause you're waiting for the phone and you've got to do something, which, you know, I think is a good thing, in that you've got to do something and you have to respond. You can't just put your phone in your pocket and walk away, but at night time, I think it would probably be a bit intrusive"* patient 01062T, follow up interview.

Participants reported being satisfied with the overall experience with the intervention, the availability of the intervention 24/7, and their ability to call in and leave a message (see Appendix 8). They would also recommend the intervention to other people who take prescribed medications (see Appendix 7).

Discussion

Principal Findings

A highly tailored intervention has been developed to support adherence to medication prescribed for high blood pressure and co-morbidities. The intervention has been systematically developed based on guidance from MRC framework, PPI/E and is based on

For peer review only

rigorous theory and evidence. It includes a combination of Behavioural Change Techniques (BCTs) tailored to patients' beliefs (e.g., beliefs about medications, self-efficacy, social norms, perceived regimen complexity) and prescription plan. The the pilot study suggested that the intervention content increased awareness about the necessity of medication adherence, the risk and benefits of maintaining adherent to prescribed medications, reinforced social support and habit formation, and reminded patients to take medications as prescribed. To our knowledge, this is the first medication adherence intervention delivered using automated text and voice messages that has been developed and piloted within the UK primary care setting. The intervention has been proven to be acceptable and feasible to older adults with high blood pressure or co-morbidities, and it is currently being evaluated in a randomised controlled trial.

Strengths and Limitations of this study

To date there are studies describing the systematic development of mHealth interventions, however none of them has provided a detailed description based on guidance from the MRC framework, and none has described the methods used to develop and pilot the implementation procedures within the primary care setting. Furthermore, this study has involved PPI/E in the intervention development, design and pilot, adapting a collaborative approach, which minimized the influence of the social desirability effect on the findings, and thus it provided a real-world perspective. As a consequence, the results of this intervention may be better informed and matched to patients' healthcare needs.

A strength of this study is the systematic approach to intervention development and piloting. The study used a rigorous theoretical approach to guide the development of the tailored intervention content. It also designed a flexible and scalable application to deliver messages to support adherence to different type, and complex prescribed medications. Another strength of this study is the utilisation of a mixed (e.g., qualitative, quantitative) and multi-perspective (patients, health care providers, PPI, stakeholders) methods of data collection. The data were combined complementarily into one comprehensive analysis, using a rigorous analytical approach.

Although this intervention is highly tailored, and participants can provide real time input and further tailor intervention content and delivery, a limitation of the intervention is the

For peer review only - http://bmjopen.bmj.com/site/about/guidelines.xhtml

self-reported theoretical determinants of medication adherence. Future studies could enable the use of objectively measured theoretical determinants and objectively measured behaviour, using sensing technology. Another constrain of the text and voice messaging is its limited features to facilitate delivery of BCTs visually (e.g., picture of tablets, graphs to show levels of adherence) [30], and future studies could usefully explore whether and how these features could influence intervention engagement and behaviour change.

Future Research

The one-month intervention has been extended to three months intervention and targets all adults (above 18 years old) to increase reach. It also includes another level of tailoring, where participants select between two BCTs to support maintenance of medication taking (e.g., habit formation or self-monitoring). The intervention is currently being evaluated in an RCT, which includes objective behavioural (medication event monitoring system) and clinical (systolic blood pressure, HbA1c) outcome measurements to inform the results about the efficacy of the intervention (see: https://doi.org/10.1186/ISRCTN10668149).

Funding statement: This paper presents independent research funded by the National Institute for Health Research (NIHR) under its School of Primary Care Research [grant number: RG86506] and the Research for Patient Benefit programme [grant number PB-PG-0215-36032]. The views expressed are those of the authors and not necessarily those of the NHS, the NIHR or the Department of Health. The funder had no role in study design, data collection, data analysis, data interpretation, the writing of the manuscript, and decision to submit the manuscript for publication.

Acknowledgment: This paper presents independent research funded by the National Institute for Health Research (NIHR) under its School of Primary Care Research [grant number: RG86506] and the Research for Patient Benefit programme [grant number PB-PG-0215-36032]. The views expressed are those of the authors and not necessarily those of the NHS, the NIHR or the Department of Health. The funder had no role in study design, data collection, data analysis, data interpretation, the writing of the manuscript, and decision to submit the manuscript for publication.

For peer review only

Author's contributions: AK conceived and designed the studies, developed the intervention, and led the intervention piloting, data collection, analyses, and wrote this manuscript. VH assisted in data collection. SE and JB provided IT expertise in developing the digital platform. SS provided guidance and consultation to AK during all stages of the intervention development and piloting process.

Conflict of interest: the authors declare no conflict of interest.

Ethics approval and consent to participate: The Office for Research Ethics Committees of Northern Ireland (ORECNI; REC references: 16/NI/0150) and Essex East of England (REC Reference number 17/EE/0203), and the Health Research Authority, have reviewed and approved this project.

Data Sharing Statement. Qualitative and quantitative data are available upon request to AK.

References

1. NICE. *Hypertension in adults: diagnosis and management.* Available from: https://www.nice.org.uk/guidance/cg127
2. The Emerging Risk Factors Collaboration. *Association of Cardiometabolic Multimorbidity With Mortality.* Jama, 2015. **314**(1): p. 52-60.
3. Public Health England. *Tackling high blood pressure. From evidence into action*. 2014; Available from: https://www.gov.uk/government/publications/high-blood-pressure-action-plan.
4. Sackett D. *Compliance with therapeutic regimens*. 1976. The John hopkins University. Baltimore & London.
5. Mazzaglia G, Ambrosioni E, Alacqua M, Filippi A, Sessa E, Immordino V, et al., *Adherence to antihypertensive medications and cardiovascular morbidity among newly diagnosed hypertensive patients.* Circulation, 2009. **120**(16): p. 1598-605.
6. Chowdhury R, Khan H, Heydon E, Shroufi A, Fahimi S, Moore C, et al. *Adherence to cardiovascular therapy: a meta-analysis of prevalence and clinical consequences.* Eur Heart J, 2013. **34**(38): p. 2940-8.

For peer review only - http://bmjopen.bmj.com/site/about/guidelines.xhtml

7. Cassell A, Edwards D, Harshfield A, Rhodes K, Brimicombe J, Payne R, et al. The epidemiology of multimorbidity in primary care: a retrospective cohort study. Br J Gen Pract, 2018.

8. NCD Risk Factor Collaboration. *Worldwide trends in blood pressure from 1975 to 2015: a pooled analysis of 1479 population-based measurement studies with 19.1 million participants.* Lancet, 2017. **389**(10064): p. 37-55.

9. NICE. *Medicines Optimisation: The Safe and Effective Use of Medicines to Enable the Best Possible Outcomes*. 2015, National Institute for Health and Care Excellence (UK)

10. NICE. *Medicines Adherence: Involving Patients in Decisions About Prescribed Medicines and Supporting Adherence*. 2009, Royal College of General Practitioners (UK)

11. Tsoli S, Sutton S, Kassavou A. *Interactive voice response interventions targeting behaviour change: a systematic literature review with meta-analysis and meta-regression* BMJ Open 2017;0:e018974. doi:10.1136/bmjopen-2017-018974

12. Kassavou A, Sutton S. *Automated telecommunication interventions to promote adherence to cardio-metabolic medications: meta-analysis of effectiveness and meta-regression of behaviour change techniques.* Health Psychol Rev, 2017: p. 1-18. doi: 10.1080/17437199.2017.1365617.

13. Smith DH, O'Keefee-Rosetti M, Owen-Smith AA, Rand C, Tom J, Vupputuri S, et al. *Improving Adherence to Cardiovascular Therapies: An Economic Evaluation of a Randomized Pragmatic Trial.* Value Health, 2016. **19**(2): p. 176-84.

14. Burn E, Nghiem S, Jan S, Redfern J, Rodgers A, Thiagalingam A, et al. *Cost-effectiveness of a text message programme for the preventin of recurrent cardiovascular events*. Heart, 2017. **103**: p. 923-930.

15. Yardley L, Morrison L, Bradbury K, Muller I. *The person-based approach to intervention development: application to digital health-related behavior change interventions.* J Med Internet Res, 2015. **17**(1): p. e30.

16. Mummah SA, King AC, Gardner CD, Sutton S. *Iterative development of Vegethon: a theory-based mobile app intervention to increase vegetable consumption.* Int J Behav Nutr Phys Act, 2016. **13**: p. 90.

For peer review only - http://bmjopen.bmj.com/site/about/guidelines.xhtml

17. Michie S, Atkins L, West R. *The Behaviour Change Wheel. The guide to developing interventions.* 2014, London

18. Craig P, Dieppe P, Macintyre S, Michie S, Nazareth I, Petticrew M, et al., Developing and evaluating complex interventions: the new Medical Research Council guidance. Bmj, 2008. 337: p. a1655.

19. Dijkstra A, De Vries H. *The development of computer-generated tailored interventions.* Patient Educ Couns, 1999. **36**(2): p. 193-203.

20. Kreuter MW, Farrell D, Olevitch L, Brennnan L. *Tailoring Health Messages. Customizing communication with computer technology*. Lawrence Erlbaum Assoc, 1999.

21. Barber N. *Should we consider non-compliance a medical error?* Qual Saf Health Care, 2002. **11**(1): p. 81-4.

22. Kassavou A, Sutton S. *Developing and pre-testing a tailored interactive voice response (IVR) intervention to support adherence to anti-hypertensive medications.* 2016, research protocol, The University of Cambridge.

23. Michie S, Richardson M, Johnston M, Abraham C, Francis J, Hardeman W, et al., *The behaviour change technique taxonomy (v1) of 93 hierarchically clustered techniques: building an international consensus for the reporting of behaviour change interventions.* Ann Behav Med, 2013. **46**(1): p. 81-95.

24. Farmer A, Hardeman W, Hughes D, Prevost AT, Kim Y, Craven A, et al. *An explanatory randomised controlled trial of a nurse-led, consultation-based intervention to support patients with adherence to taking glucose lowering medication for type 2 diabetes.* BMC Fam Pract, 2012. **13**: p. 30.

25. Kassavou A, Sutton S. *Reasons for non-adherence to cardiometabolic medications, and acceptability of an interactive voice response intervention in patients with hypertension and type 2 diabetes in primary care: a qualitative study.* BMJ Open, 2017. **7**(8): p. e015597.

26 Wilkinson S. *Qualitative data collection: interviews and focus groups*, in *Research methods for clinical and health psychology*, Yardley, L., Marks D, Editor. 2004. Sage Publications Ltd.

For peer review only

27. Alexander V, Thomas H, Cronin A, Fielding J, Moran-Ellis J. *Mixed methods* in *Researching Social Life*, N. Stoneman, Editor. 2016: London p. 119-138.

28. Moran-Ellis J. *Following a Thread – An Approach to Integrating Multi-method Data Set.'* Paper given at ESRC Research Methods Programme, Methods Festival Conference, Oxford, July 2004.

29. O' Cathain A, Murphy E, Nicholl J. Three techniques for integrating data in mixed methods studies. BMJ, 2010; 341:c4587.

30. Armitage L, Kassavou A, Sutton S. A systematic review of the literature with meta analysis and meta regression: the use of smartphone applicatons to suport medication adherence. Prospero protocol, 2018.

Appendix 1. Intervention development process, informed by the MRC Framework

Appendix 2. Intervention piloting process, informed by the MRC framework

Appendix 3: Map of the theoretical framework, determinants and BCTs to produce changes in adherence to medications.

Appendix 4: PPI/E messages

Appendix 5: Example of message tailoring.

Appendix 6. Participants' demographic characteristics

Appendix 7. Participants' perception of the intervention utility and future use

Appendix 8. Participants' satisfaction with the intervention components.

For peer review only

Appendix 1. Intervention development process, informed by the MRC Framework.

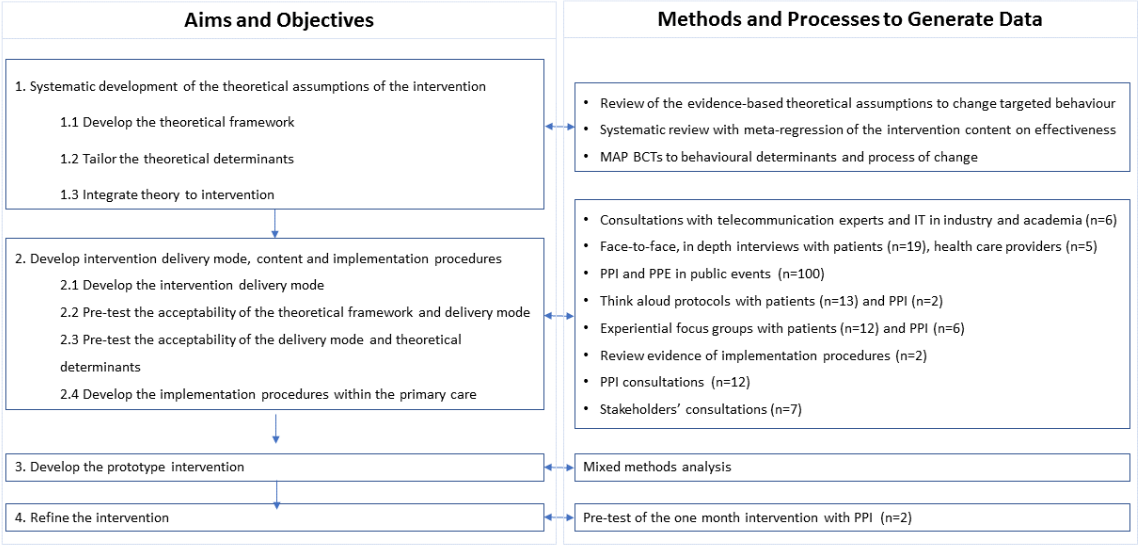

For peer review only

Appendix 2. Intervention piloting process, informed by the MRC framework.

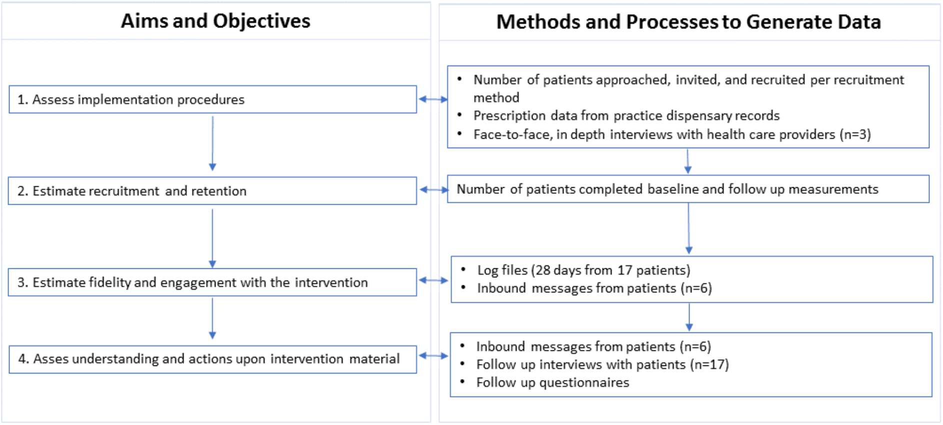

r review only

Appendix 3. Map of the BCTs to theoretical determinants and framework.

	Framework	Theoretical Determinant	BCTs
PRIMARY DETERMINANTS	INA	**Beliefs and knowledge**	*Re-attribution*
			Pros and cons
			Information about antecedents
			social comparison
			reduce negative emotions
			normative information
		**outcome expectations**	*Information about health consequences*
			information about emotional consequences
			Information about social and environmental consequences
			Pros and cons
			goal setting (behaviour)
			goal setting (outcome)
			self talk
			Social support (unspecified)
			Social support (practical)
			Socia support (emotional)
		**self efficacy**	*feedback on behaviour*
			reporting whether or not the behaviour was performed
			Problem solving
			action planning/ implementation intentions
			self monitoring of behaviour
			adding objects to the environment
			feedback on outcomes of behaviour
			biofeedback
			review behaviour goals
	NINA		*review outcome goals*
			feedback on emotional state
			monitoring of emotional consequences
			social reward
			Social support (unspecified)
			Social support (practical)
			Socia support (emotional)
			self talk
		**habit**	*Prompts/cues*
			habit formation
			action planning/ implementation intentions
			feedback on behaviour
			Social reward
SECONDARY DETERMINANTS		**social support**	*social comparison*
			social reward
			Social support (unspecified)
			Social support (practical)
			Socia support (emotional)
		**emotional/ affective state**	*Information about emotional consequences*
			Report emotional state
			Feedback on emotional state
			Monitoring of emotional consequences

Appendix 4. Examples of Patient and Public Involvement and Engagement (PPI/E) messages.

**Scenario:** Mark is a 49 year old car mechanic. He was diagnosed with high blood pressure by his GP 6 months ago even though he had no symptoms, was not overweight, did not smoke, ate healthily and had an active job. Initially, he took the medication exactly as his GP prescribed every day. However, after a few weeks he began to experience constipation and read on the leaflet supplied with the tablets that this was a side effect caused by the medication. Now, he only takes his medication every other day and the constipation has cleared up. However, his blood pressure is still too high.	
**Message**	**Event**
Keep on the tablets prescribed to you by your Doctor. High blood pressure is more serious than constipation.	HOP
His action was foolish – he should have consulted his GP before changing the prescribed medication.	HOP
This scenario similar to mine. However I do take my meds without fail. I am motivated by my family history which leads me to believe I may be susceptible to heart disease which, of course, is thought to be related to blood pressure. I can understand why people might be concerned about side effects, so they need motivation.	HOP
I know that mechanics are frequently asked to work through lunch breaks etc. Please take some fruit and water to work with you to snack on throughout the day. This will help with your constipation, as you should be taking your pills every day. Good luck.	HOP
You need to talk to your GP in order for him to prescribe a laxative to help with the constipation. The need to take your BP medication is crucial in bringing your BP down.	HOP
BP tablets must be taken every day so consult your GP for medication for constipation.	HOP
It's stupid not to take your meds! If you're constipated just eat more fibre. It's not worth the risk.	HOP
Hey Mark. Are you aware of your blood pressure today? Have you taken your meds yet?	SF
Hi Mark. I sympathise a lot. I used to suffer from blood pressure and constipation, but nowadays I rarely suffer from either. I've had heart surgery but still cycle a lot. Don't give up on the meds. There are different tablets available: ask your GP. But also, use whole brand cereals regularly, even at bedtime!	SF
Hi Mark. You've sorted the constipation – keep going for the blood pressure!	SF
Mark, you should keep taking your medication even with the side effects, because it is better for you.	SF

Abbreviations: Hospital Open Event – HOP; Science Festival – SF; Focus Groups – FG

For peer review only

Appendix 5. Examples of tailoring messages.

Tailored theoretical determinants	BCT	Message file
↑ NINA and ↑ emotional state	Habit formation	Please do not forget to take your tablets. To achieve better control over high blood pressure, you will need to take them every day.
NINA / INA and ↓ social norms	Report whether or not the behaviour was performed, social reward, and information about health consequences	IVR: We are calling to see how you are doing with taking your tablets. Have you taken all your tablets as prescribed today?<br>P: yes<br>IVR: Well done! Taking your medications as prescribed is an easy way to keep blood pressure down.
↑ NINA	Habit formation	You are soon due to take your tablet. Please do not forget to do so.
NINA / INA and prescription plan	Habit formation and action planning	This is your message for your blood pressure tablets. One easy way to remember your tablets, is to take them with another daily activity, such as your morning cup of tea. If they are always done together, it will be harder to forget.
↑ Beliefs about medications, ↑ self-efficacy to take medications when no health benefits and ↑ emotional state	Habit formation and information about emotional consequences	Please keep taking your tablets as prescribed even if you are well and feeling healthy. High blood pressure is one of those things that unless you actually feel it you're not aware that it is a problem. Taking your medications as prescribed will support you to keep enjoying things or activities that are important to you.
NINA, ↓ beliefs about medications and ↓ emotional state	Habit formation and social support (unspecified)	Whatever the day may holds, please do not forget to take your tablets. To achieve better control over high blood pressure, you will need to take them every day.
↓ self-efficacy to cope with ↓ practical social support	Habit formation and social support (unspecified)	We are calling to see how you are doing with taking your tablets today. Please remember that you can always call the free telephone number if you require more support on how to take your tablets as prescribed. If you require more information about the health condition or the dosage of your medications, please contact your GP practice or pharmacist.
↑ social norms and ↑ self-efficacy to cope with ↓ practical social support	Habit formation and social support (unspecified)	You may find it helpful to ask people who are important to you to remind you to take your medications as prescribed and maintain your blood pressure under control. Please remember that you can always call the free telephone number if you require more support on how to take your tablets as prescribed.

Note: ↑indicate higher score from the cut-off point, ↓ indicate lower score from cut-off point.

For peer review only

Appendix 6. Participants' demographic characteristics

Age		Work status		Health condition		Gender	
80-89	7.8%	Retired	76.5%	Hypertension	64%	Male	65.7%
70-79	28.1%	Part-time	10.9%	Type 2 Diabetes	14%	Female	34.3%
60-69	43.7%	Full time	10.9%	Co-morbidities	22%		
50-29	15.6%	Unable to work	1.7%				
40-49	4.6%						

Note: results from N=64 patients taking part in intervention development studies (interviews n=19; think aloud protocols n=13; focus groups n=12; consultations n=2) and pilot study (n=18).

For peer review only

Appendix 7. Participants' perception of the intervention utility and future use

	Mean (sd)
The intervention messages helped me to take my tablets as prescribed	3.2 (1.3)
The intervention reminded me to take my medications as prescribed	3.5 (1.3)
The intervention was easy to use	4.3 (0.7)
The intervention addressed my personal needs	3.3 (1.1)
It would have been useful, if the intervention was part of the regular care	3.7 (0.9)
I would recommend the intervention to other people who take medications for long-term health conditions	4.2 (0.8)

Note: Likert scale, 1 strongly disagree to 5 strongly agree. Data from n=17 participants.

For peer review only

Appendix 8. Participants' satisfaction with the intervention components.

	Mean (sd)
The experience with the intervention	4 (0.7)
The voice delivering the intervention messages	3.7 (1.1)
The content of the intervention messages	3.8 (0.8)
Being able to call the intervention	4 (0.7)
The availability of the intervention 24/7	4.2 (0.8)
The response you received from the intervention	3.4 (0.7)
The response you received to your particular queries/questions	3.4 (0.7)

Note: Likert scale, 1 very dissatisfied to 5 very satisfied. Data from n=17 participants.

For peer review only

BMJ Open

Development and piloting of a highly tailored digital intervention to support adherence to anti-hypertensive medications, as an adjunct to primary care consultations.

Journal:	*BMJ Open*
Manuscript ID	bmjopen-2018-024121.R2
Article Type:	Research
Date Submitted by the Author:	17-Oct-2018
Complete List of Authors:	Kassavou, Aikaterini; University of Cambridge, Public Health and Primary Care ; University of Cambridge,<br>Houghton , Vikki<br>Edwards, Simon<br>Brimicombe, James; University of Cambridge, Primary Care Unit, Department of Public Health and Primary Care<br>Sutton, Stephen; University of Cambridge,
**Primary Subject Heading**:	Public health
Secondary Subject Heading:	Cardiovascular medicine, Health services research, Public health
Keywords:	medication adherence, Hypertension < CARDIOLOGY, interactive voice response, text messages, pilot intervention

SCHOLARONE™
Manuscripts

INTERVENTION DEVELOPMENT AND PILOTING

Development and piloting of a highly tailored digital intervention to support adherence to anti-hypertensive medications, as an adjunct to primary care consultations.

Aikaterini Kassavou, Vikki Houghton, Simon Edwards, James Brimicombe, and Stephen Sutton

Address: University of Cambridge, School of Clinical Medicine, Department of Public Health and Primary Care, Forvie Site, Robinson Way, Cambridge, CB2 0SR

Corresponding author: Aikaterini Kassavou, University of Cambridge, School of Clinical Medicine, Department of Public Health and Primary Care, Forvie Site, Robinson Way, Cambridge, CB2 0SR.

Email: kk532@medschl.cam.ac.uk

Key words: medication adherence, hypertension, interactive voice response, text messages.

Word count: 4911

For peer review only

Abstract

Objectives: This paper describes the systematic development and piloting of a highly tailored text and voice message intervention to increase adherence to medication within primary care. Methods: following the MRC guidance this paper describes: a) the systematic development of the theoretical framework, based on review of theories and meta-analyses of effectiveness; b) the systematic development of the delivery mode, intervention content and implementation procedures, based on consultations, face-to-face interviews, think-aloud protocols, focus groups, systematic reviews, PPI/E input, intervention pre-test, and c) the piloting of the intervention, based on a one-month intervention; and follow up assessment including interviews and questionnaires. The mixed methods analysis combined findings from the parallel studies complementarily.

Results: intervention development suggested the target behaviour of the intervention should be the tablets taken at a regular time of the day. It recommended that patients could be more receptive to intervention content when they initiate medication taking or they change prescription plan; and more emphasis is needed to patients' consent process. Intervention piloting suggested high intervention engagement with, and fidelity of, the intervention content; which included a combination of BCTs, and was highly tailored to patients' beliefs, and prescription plan. Patients reported that the intervention content increased awareness about the necessity to take and maintain adherent to medication, reinforced social support and habit formation, and reminded them to take medication as prescribed.

Conclusion: tailored automated text and voice message interventions are feasible ways to increase medication adherence, as an adjunct to primary care.

Keywords: adherence, anti-hypertensive medication, text messaging, interactive voice response.

For peer review only

For peer review only - http://bmjopen.bmj.com/site/about/guidelines.xhtml

INTERVENTION DEVELOPMENT AND PILOTING

Strengths and limitations of this study

- This is the first medication adherence intervention for patients with hypertension and co-morbidities that has been developed and piloted within the UK primary care setting.
- The study used rigorous methodology to collect and analyse data, from multiple perspectives.
- This study involved and engaged Patients and Public at all stages of the intervention development and piloting.
- Future studies should include real-time and objective measures of the intervention determinants and outcomes.

For peer review only

For peer review only - http://bmjopen.bmj.com/site/about/guidelines.xhtml

INTERVENTION DEVELOPMENT AND PILOTING

Introduction

Hypertension or high blood pressure (HBP), affects approximately one in four people in England [1], and often accompanies other conditions, including type 2 diabetes mellitus (T2DM), coronary heart disease and stroke [2]. These conditions are major risk factors for disability and premature death [2], and medication adherence can significantly lower these risks [1]. For example, in England over ten years, an estimated 7,000 quality of life years (QALYs) could be saved and £120m not spent on related health and social care, if people had better adherence to anti-hypertensive medications [3].

Adherence is defined as taking at least 80% of the prescribed tablets, and/or having medications dispensed for at least 95% days of a prescribed period [4]. However, substantial proportions of patients do not take their medication as prescribed [5]. A recent meta-analysis showed that 40% of people do not adhere adequately to cardiovascular medication and the percentage was similar (41%) for non-adherence to anti-hypertensive medications [6]. Taking into consideration the growing prevalence of HBP, co-morbidities and the ageing population [7], especially in lower and middle-income countries [8], it is likely that there will be an increase in demand for health care resources to support medication adherence. The National Institute of Health and Care Excellence (NICE) recommends the development of novel and cost-effective interventions to aid patients' decision making about taking medicines, as an adjunct to health care providers' consultations [9, 10].

Tailored mHealth interventions, such as text (SMS) and voice (IVR) messaging intervention, is one way to support patients' adherence between their consultations. Review evidence found that such interventions can effectively support adherence to different types of medication, including antihypertensive tablets, when compared to usual care only [11, 12], and can potentially be cost-effective if applied to a large number of people [13]. For example, a recent study suggested that such interventions could result in a 2.3% increase in QALYs and overall savings of 21,120 Australian dollars during a patient's lifetime [14]. However, no such intervention has been developed and piloted in the UK primary care setting.

The aim of this paper is to describe the development and piloting of a highly tailored text and voice messaging intervention to support adherence to antihypertensive tablets. Although several guidelines have been proposed to the development of mHealth interventions [15-17], this paper has been informed by the Medical Research Council (MRC) Framework [18] as it leads not only the process to develop the intervention content and delivery mode, but also the

For peer review only

implementation procedures within the primary care National Health Service (NHS), which is an important process in developing and piloting novel interventions.

Methods

Review of theory and evidence, interviews, focus groups, consultations and Patients and Public (PPI) involvement and Engagement (PPE) informed the intervention development process (see Appendix 1); and a one-month pre-post intervention study, with follow up assessment informed the piloting of the intervention (see Appendix 2).

Intervention Development

Step 1. Systematic Development of the Tailored Intervention.

The systematic development of the tailored intervention was informed by the guidance provided by Dijkstra and De Vries [19] and Kreuter and colleagues [20] on the process of developing computer telephony interventions. This process included the development of the theoretical framework, tailoring, and integration.

Develop the theoretical framework. The theoretical framework that distinguishes between intentional non-adherence (INA) and non-intentional non-adherence (NINA) guided the development of this intervention [21]. To address INA and NINA, we identified the empirical evidence of the proximal and modifiable determinants of adherence to medication [22] and mapped them into INA and NINA framework. Following that, we used the Taxonomy of the Behaviour Change Techniques (BCTs) [23] to identify the techniques that impact on INA and NINA determinants (see Appendix 3). Our decision on the BCTs for this intervention was informed by findings from two systematic literature reviews [11, 12] and previous empirical evidence [24].

Tailor the theoretical determinants. To develop a highly tailored intervention we produced a tailoring matrix, which consists of a theory-based questionnaire, an algorithm of decision rules, the schedule and the message file. The theory-based questionnaire includes items measuring each participant's values of the theoretical determinants. The algorithm includes decision rules (i.e., cut-off points), which facilitates the decisions on what message to prioritize and indicates a default feedback. The tailoring process of the intervention messages is informed by multiple sources, which can be grouped in three 'clusters': the first cluster of tailoring is based on participant's unique characteristics (e.g., name) and is informed by records from the primary care practices; it aims to gain person's attention to the intervention by making messages personally relevant. At the second cluster of tailoring we use data from participant's

For peer review only - http://bmjopen.bmj.com/site/about/guidelines.xhtml

responses at the tailoring questionnaire and their prescription plan. This cluster includes two sub-levels of tailoring: tailoring based on the degree of (a) INA and NINA, and (b) each of the modifiable INA and NINA theoretical determinants. At the third cluster, we tailor the intervention to participants' responses and feedback during the intervention. Participants are able to further tailor the intervention content by requesting a higher or lower degree of information for a particular determinant or by requesting feedback on a different determinant. Intervention tailoring at this cluster takes into consideration participants' navigation options until the request. The schedule includes the frequency of the messages for the duration of the intervention, which participants can increase, reduce or stop. The message file included the tailored BCTs.

Integrate theory to intervention. The frequency, combination and sequence of the BCTs were integrated into one intervention. The decision on the above elements was based on the phase participants are in (e.g., uptake or maintain), having as the starting point the collection of the medication from the dispensary.

Step 2. Development of the Intervention Delivery Mode, Content, and Implementation Procedures.

This step of the intervention development aimed to convert the theoretical underpinnings into features of the delivery mode, intervention content, and implementation procedures, and it involved: the development of the delivery mode, the pre-test of the theoretical framework and the delivery mode, the pre-test of the delivery mode and the theoretical determinant, and the development of the implementation procedures within primary care.

Develop the intervention delivery mode. To translate the theoretical underpinning into features of the intervention delivery mode, consultations with six experts from the industry and academia were conducted. Meetings with telecommunication and IT experts in the University of Cambridge informed the decisions about the development of the IVR application. The discussions informed the development of features of the IVR applications, which involved the navigation options, the degree of interactivity, the voice delivering the messages, and the prompts to facilitate participants' navigation and dialogue. The discussions for the development of the SMS application involved the necessary features to accommodate messages on complex prescription plans. A custom written database application contained the functionality to create output for both delivery modes, and data were transferred at regular intervals to both platforms (IVR and SMS) using secure data hosting protocols. The digital platform could also facilitate participants' feedback during the intervention or at a different

For peer review only - http://bmjopen.bmj.com/site/about/guidelines.xhtml

time (i.e., by triggering an inbound call). Voice recognition facilitated interaction and data collection. The digital platform enabled flexible schedule of messages delivery; for example, messages could be delivered at times tailored to participant's prescription plan (e.g., 1-4 messages per day).

Pre-test the acceptability of the theoretical framework and delivery mode. One interview study and Patients and Public Involvement (PPI) and Engagement (PPE) informed the acceptability of the theoretical framework and delivery mode.

Face-to-face, in depth interviews were conducted with patients and health care providers to assess the acceptability of the delivery mode, the theoretical framework, and generate relevant content. Patients were randomly identified and selected by the practice manager from practice databases. Primary care practices within the Cambridgeshire and Peterborough Clinical Commissioning Group (CCG) were eligible for inclusion, and five primary care practices from different areas of deprivation (based on Index of Multiple Deprivation; IMD) took part in the study. Patients were eligible if they: (a) had a diagnosis of HBP, T2DM or both health conditions; (b) had been prescribed at least one anti-hypertensive medication and/or glucose lowering medication as confirmed by practice records for at least three months before recruitment; (c) had poorly controlled blood pressure and/or glucose levels as indicated by practice records, or had gaps in collecting repeat prescriptions; and (d) were aged 18 years or older. Patients were excluded when they: (a) had hearing or speaking impairment, (b) had a diagnosis of dementia, aphasia or other cognitive difficulties that could affect the study participation, (c) had a recent severe life-threatening event, (d) had difficulty in speaking or understanding English, or (e) participated in another study. A practice GP double screened the list of selected patients against the inclusion and exclusion criteria to confirm eligibility. Eligible patients were invited by post or telephone calls following up a postage invitation. Health care providers (i.e., nurses and health care assistants) who had experience with advising patients about their medications were eligible to participate and interviewed in the practices. Nineteen patients and five health care providers were interviewed face-to-face. Both patients and health care providers' interview data informed inductively the content of the theoretical determinants and the BCTs, and provided their views on the acceptability of the automated intervention within the primary care setting.

Patients and Public Involvement (PPI) and Engagement (PPE) was conducted to assess acceptability of the delivery mode and the theoretical framework, and inform the intervention content. PPI/E members were recruited opportunistically using emails or adverts. In total 100 PPI/E members took part in three events: one meeting with a diabetes patients' group (n=20),

For peer review only - http://bmjopen.bmj.com/site/about/guidelines.xhtml

one open event at Addenbrookes' Hospital (n=30), and one open event at the Cambridge Science Festival (n=50). During the three PPI/E events, members were asked their views and recommendations on the delivery mode, and to write messages tailored to INA and NINA case studies. Data generated from PPI/E informed the content of the message file (for example, see Appendix 4). Overall, PPI/E members found more acceptable NINA case studies and seemed to generate more messages for NINA rather than INA case studies.

Pre-test feasibility of intervention delivery mode and theoretical determinants. Four studies informed the acceptability of the delivery mode and the theoretical determinants. These included: think aloud interviews, experiential focus groups, and PPI.

Face-to-face, in depth interviews were conducted with 13 patients. Think aloud protocol was used to assess reliability of the mechanisms of change (i.e., link between the theoretical determinants and the BCTs), and usability of the delivery mode. All interviews were conducted at participants' places. Field notes and think aloud data informed the analysis, and results refined the tailoring of the theoretical determinants [25].

Face-to-face meetings were conducted with two PPI members recruited using emails from the list of the National Institute of Health Research (NIHR) Cambridge BRC PPI/E Panel and Communications office. Think aloud protocol was used to assess the reliability and sequence of the items to form the measurement of the theoretical determinants (i.e., theory-based questionnaire). Both interviews were conducted at the University. Field notes and think aloud data informed the analysis, and results refined the reliability of the tailoring questionnaire and informed the decision rules of the tailoring algorithm.

Experiential focus groups aimed to obtain patients' views of, and refine, the tailored intervention content (e.g., inclusion of medication name, dose and timings; 'please do not forget to take Ramipril 1 tablet 10 mg at 9am') and delivery mode within a social interaction context [26]. Eligibility criteria and recruitment procedure were similar to patients' face-to-face interviews, reported above. Four focus groups took place in primary care practices (n=2) or community centres (n=2). During the focus groups patients (n=12) received examples of SMS and IVR messages on their mobile phones and were asked to provide experiential feedback. Patients were asked their views and recommendations about the combination of the delivery modes and the intervention content, using a think-aloud protocol [27].

One experiential focus group with six PPI members aimed to obtain views and recommendations on the intervention content and delivery mode. PPI were recruited using emails from the NIHR Cambridge BRC PPI/E office. PPI members were eligible to take part

For peer review only - http://bmjopen.bmj.com/site/about/guidelines.xhtml

if they were prescribed medications for a long-term health condition and were over 18 years old.

The data generated from the experiential focus groups informed the content of the message file and the process to map the intervention messages onto the theoretical determinants.

Develop implementation procedures within primary care. The intervention implementation procedures were informed by systematic review evidence, PPI/E, and stakeholders' consultations.

Information about the recruitment setting and patients' characteristics at baseline, as well as uptake and retention rates were extracted from the two systematic reviews of trials on medication adherence delivered by SMS and/or IVR messages [11, 12]. Due to missing data about the characteristics of the targeted population, the recruitment setting, recruitment methods and material, we synthesised the findings narratively. Results informed the decisions regarding the recruitment setting and methods, as well as the targeted behaviour and population of the intervention.

Twelve PPI members provided feedback on the recruitment material (e.g., patients' information sheets, posters, leaflets, flyers, and invitation letters) using emails or face-to-face meetings. Their comments informed the content of the recruitment material and the description of the recruitment, informed consent, and intervention procedures in lay language.

Stakeholders' consultations were conducted with health care providers, commissioners, and patients to inform the study design and the implementation procedures within the primary care setting (e.g., recruitment, informed consent, data collection). Practitioners with experience of advising patients for medications; commissioners for medicine optimisation, prescribing or cardiovascular conditions; and patients with either HBP or both HPB and T2DM were included. Commissioners were recruited informally by email through personal networks. Patients and health care providers (i.e., practice nurses, health care assistants) were recruited through the primary care practices. Those who consented to take part were invited to the consultations. Consultation modes were flexible to maximise participation (n=3 face-to-face, n= 4 email). Stakeholders (N=7) were given a description of the proposed intervention and asked their views and recommendations. Health care providers (n=4) were consulted about the time to initiate recruitment, and the recruitment methods and procedures. Commissioners (n=1) were consulted about how best to track resource use and costs and the evidence needed to inform whether or not to commission such an intervention. Patients (n= 2) were consulted about the delivery mode and the theoretical framework. All stakeholders were asked for their

For peer review only - http://bmjopen.bmj.com/site/about/guidelines.xhtml

recommendations about the implementation procedures. The data generated from stakeholders' consultations informed the study and intervention implementation procedures.

Step 3. Develop the Prototype Intervention.

A prototype intervention was developed by multiple and iterative syntheses of the data obtained during the intervention development. The intervention is highly tailored to deliver very brief (≥1 minute), theory-based messages at participant's preferred time, telephone and frequency to receive the calls. The pilot intervention consisted of 29 messages, delivered daily for a duration of one prescription-based month. Two messages were delivered at the first day of the intervention, with feedback tailored to each participant's responses at the baseline questionnaire and prescription plan, and one message per day during the following 27 days of the intervention.

Step 4. Refine the Intervention.

Two PPI members, recruited by the NIHR Cambridge BRC PPI/E office, pre-tested the prototype intervention for one prescription-based month. PPI provided their experiential feedback about the intervention usability and mechanisms of change during four, weekly telephone-based interviews with a researcher, and completed the follow up questionnaire. The data informed the decision rules of the tailoring algorithm and refined the frequency (i.e., message schedule), combination and sequence of the BCTs (i.e., integration of theory into the intervention), before piloting the prototype intervention within primary care.

Intervention Piloting

A one-month pilot intervention was conducted to assess (a) implementation procedures, (b) uptake, retention, fidelity, and engagement with the intervention, (c) participants' views, understanding, and actions upon the intervention content; and obtain (d) recommendations for improvement.

Patients with HBP were recruited by primary care practices within the Cambridgeshire and Peterborough CCG, and health care providers who consulted patients for taking medications. Patients were selected and recruited using the same recruitment procedure described in the intervention development studies but included two additional recruitment strategies: (a) face-to-face recruitment by health care providers, and (b) leaflets and posters in the practice waiting rooms. Health care providers received training by a researcher on the face-to-face recruitment

For peer review only - http://bmjopen.bmj.com/site/about/guidelines.xhtml

and signposting procedures. Patients completed measures at baseline (T1) and at the end of the intervention (T2).

Patients completed the tailoring questionnaire (T1, T2), their preferred primary and secondary telephone number to receive the intervention messages, and the time intervals of repeated calls per telephone number (T1), two items measuring adherence (T1, T2), 16 items measuring their experience and satisfaction with elements of the intervention (T2), and face-to-face interviews with a member of the research team (T2). Medication collection data for the duration of three months before T1, and at T2, were obtained from practice dispensary records. Telephone log files assessed uptake, retention, fidelity, and engagement with the intervention. Participants' inbound calls assessed intervention engagement and impact. Face-to-face interviews (T2) assessed users' understanding and actions upon the intervention and obtained recommendations for improvement. Face-to-face interviews with health care providers (n=3) assessed implementation procedures and obtained recommendations for improvement.

Analysis

Data generated from the intervention development and pilot study were integrated into one mixed methods analysis [28], using the technique called "following a thread" [29, 30]. The theoretical underpinnings of the intervention were explored across different methods of data collections, data generated from each method informed each intervention component (i.e., intervention content and implementation procedures) iteratively, and questions or contradictory findings generated were followed across the other methods until saturation was achieved.

Patient and Pubic Involvement. PPI/E was included in all parts of intervention development and piloting.

Results

A summary of the outcomes of the mixed methods analysis are reported in table 1, and explained in more details at this section

Table 1. Outcomes and results from intervention development and piloting.

**Intervention development**	**Intervention piloting**
• Theory based questionnaire<br>• Tailoring algorithm	• Recruitment and retention rates

For peer review only - http://bmjopen.bmj.com/site/about/guidelines.xhtml

INTERVENTION DEVELOPMENT AND PILOTING

• Message file<br>• Message schedule<br>• Delivery mode<br>• Targeted behaviour and participants' characteristics<br>• Implementation procedures within the primary care	• Fidelity and engagement with the intervention<br>• Understanding and actions upon intervention material

Theory Based Questionnaire

Participants' feedback on the questionnaire suggested that self-reported measures of non-adherence have confounded effect on measures of psychological constructs of adherence, and thus an objective measure of adherence was recommended. For example, Medication Adherence Report Scale items were perceived to measure INA/NINA constructs, rather than taking or not taking medications.

Tailoring

Participants' input suggested amendments to the tailoring algorithm, to increase the sensitivity of the algorithm in selecting INA participants. For example, the cut-off points of the algorithm were amended to facilitate the identification of INA and NINA. Moreover, participants' input suggested that the theoretical determinants could be the same for INA and NINA but they might differ in terms of the value of tailoring, thus we adjusted the theoretical model and cut-off points, so that each of the theoretical determinant could appear to both INA and NINA participants (for example, see Appendix 5, for examples of tailoring BCTs).

Message File

Participants suggested content that informed the intervention BCTs, so that we included more BCTs, such as information about social and environmental consequences.

> "*Do you, sort of, remind them of the costs and things? Is that in with medication and things, if they're missing it, or every time they lose blood pressure medication or throw it in the bin, what is the cost?* " Practitioner consultation

Schedule

The one-month intervention schedule included daily messages and participants could change the frequency or stop them. Patients at follow up interviews, recommended an intervention of longer duration with messages to gradually decrease in frequency and include advice and support for self-monitoring medication taking.

Targeted Behaviour and Participants' Characteristics

For peer review only - http://bmjopen.bmj.com/site/about/guidelines.xhtml

INTERVENTION DEVELOPMENT AND PILOTING

Patients reported that it was easier for them to report on the pills taken per day, rather than the specific type of tablets (e.g., felodipine, statins). However, they reported that this information could be useful for patients who initiate medication taking, and need more education about their prescribed regimens. Patients reported that the timing of medication-taking or the routine associated with the timing (e.g., lunch), rather than the type of medications to take, was an easier reminder of behaviour. That was particularly important for those patients taking medication for longer and for more than one health conditions.

> Q: "*How many tablets do you take per day?*" R*: "Oh, about 17"*. Q: *"How many different types of medication do you take per days?"* R: *"Yeah. That takes more counting and a bit more thinking ... I suppose, as well, if you have so many, you just get used to the times…Morning, lunch, evening, night time*" patient

Patients who took part in the intervention development studies and the pilot study were recruited from different areas of IMD, were older adults, had either HBP or co-morbidities (see appendix 6, for participants' demographic characteristics), and self-reported being prescribed complex medication regimens (number of tablets per day: mean=6, sd=4.6).

Implementation Procedures within Primary Care

Health care providers that conducted the recruitment procedures reported challenges with recruiting non-adherent patients and recommended recruitment strategies delivered at times when patients might be more receptive to uptake such an intervention. In accordance to the systematic review evidence, it was recommended that patients would be more receptive to uptake the intervention when they initiate medication taking or when there is a change to their current prescription plan (e.g., this could be either due to a health event and/or due change to the pharmaceutical product). Otherwise, patients could be reluctant to provide consent and uptake the intervention (table 2).

Table 2. Health care providers' experiential feedback

Theme	Quote
Views about recruiting non-adherent patients	*"[patients who do not adhere to medication] they're just not coming in for their monitoring ... a lot of the names on the list [of eligible participants] were people that weren't coming in anyway and that was the biggest problem*" practice nurse
Views about barriers to recruit for a digital intervention	"*cause they thought it was them, they had bad blood pressure or something ... there was a question about what other medication do you take as well, and they're like, 'Why do we have to answer that if it's only about the blood pressure one?' ... but he felt like they were invading his space, sort of thing, by putting down all the medications, you can kind*

For peer review only

INTERVENTION DEVELOPMENT AND PILOTING

	of guess what they're for, and that's what he didn't really want to" practice nurse
Views and recommendations about recruitment methods	Annual reviews: "*annual review is a bit more time … when its annual reviews because we're actually talking about their tablets*" practice nurse recommendation about recruitment procedures<br>Practice website: "*but you can put a facility on the online forum. A lot of people access our website, so there are quite a lot of other places that people might look maybe more than a poster*" practice nurse<br>Medication prescriptions: "*but every time you get a repeat prescription list, you can write something on it*" practice nurse

Note. N=8 health care providers: nurses and health care assistants. Themes have been coded at interviews with health care providers at development (n=5) and piloting of the intervention (n=3). Quotes reported in table are from experiential interviews with health care providers (T2).

For peer review only

Health care providers recommended that recruitment methods could be integrated to annual reviews or medication reviews. They also recommended that non-adherent patients, who do not attend the practice for their annual or medication reviews, either because they do not appreciate the need of these or they are sceptical about the recommended treatment, could be more receptive to the intervention if invited by other methods; like notes into their prescriptions, text messages, newsletters, or multimedia (table 2).

However, health care providers also revealed that time constrains prevented them from explaining the implementation procedures to eligible patients during primary care consultations. It also prevented them from facilitating patients' motivation to medication adherence and uptake to the digital intervention.

In line with data from the PPI consultations and patients' interviews, health care providers suggested that there is a lot of information in the recruitment material and suggested that more emphasis is needed to patient's consent process. It was suggested that patients should be made aware about what information will be collected by their practice records, and how these will be utilised from the intervention. To facilitate patients' understanding and standardise the implementation procedures, including the informed consent, we developed videos aligned to written material. The content of the videos aimed to also provide normative information about taking medication and medication non-adherence, and increase motivation to initiate behaviour change. The videos were integrated into the study invitation material (e.g., text message invitations to eligible patients) and brief consultations with the health care providers.

To reinforce the uptake and integration of the intervention into current NHS, commissioners, patients, and health care providers reported that intervention needs to be of low or no cost. *"Would need to be very low cost or free to use"* commissioner consultation.

For peer review only - http://bmjopen.bmj.com/site/about/guidelines.xhtml

INTERVENTION DEVELOPMENT AND PILOTING

Intervention Uptake and Retention

Twenty patients were recruited. Eighteen provided written informed consent and registered into the study, of whom 17 completed the one-month intervention: one patient was excluded by the research team before registration because he/she met one of the exclusion criteria, one patient withdrew before registration to the intervention, and the other participant dropped out during the intervention. All 17 patients completed the measures at baseline and at the end of the intervention. All primary care practices provided completed baseline (e.g., refill prescription data three months before the start of the intervention) and follow up data, for all participants.

Intervention Fidelity and Engagement

During the one-month intervention an average of 29 messages were scheduled, of which 22.52 were received by patients. On average, 37 calls were made, of which 2.8 failed to be made due to technical issues and 13 calls were made but failed to go through because they were not picked up by the participants. On average, three calls were repeated and received by participants. Ten out of the 17 participants had chosen the calls to be repeated if not answered, and five out of the 17 participants had provided a secondary number for the calls to be repeated, if there was no answered at the primary number. Overall, patients were engaged with the intervention and made inbound calls to change the schedule of the messages or report about intervention content. In summary, there was a good fidelity of the intervention BCTs, with the majority of the BCTs having high fidelity scores (table 3).

Table 3. Fidelity and engagement, intervention content

Messages, including one or more BCTs	*Scheduled*	*Received*
Tailored to baseline questionnaire, integration of tailored BCTs	1	0.6
Personalised	29	22.52
Information about health consequences	5	4.41
Information about emotional consequences	1	0.65
Action planning, implementation intentions	4	4
Report whether or not the behaviour was performed	2	1.74
Social reward	2	1.4
Habit formation	24	19.58
Social support (unspecified)	3	2.29

Note. N=17 patients. Data reported as average number. The average number of the single BCTs reported in this table, excludes the number of BCTs included in the "tailored to baseline questionnaire, integration of tailored BCTs".

Understanding and Actions upon Intervention Content

For peer review only

INTERVENTION DEVELOPMENT AND PILOTING

Participants reported that the intervention content increased their awareness about medication adherence and the risk and benefits of maintaining adherent to prescribed medications for long term, reinforced social support and habit formation, and reminded them to take medications as prescribed (table 4, Appendix 7).

Table 4. Participants' experiential feedback about the intervention content

Elements of the Intervention	Themes and quotes
Message content and tailoring<br><br>*Understanding and acceptability of the theoretical determinants*	Increase awareness of the importance to take medications as prescribed<br>"*So, that message came over quite clear, you know, that you must – you mustn't miss them. You must take them, you know. So, I thought it was quite good.*" patient 01001X<br>Increase awareness of the benefits to keep taking medications as prescribed, and potential risks when not taking medications<br>Q. *Can you remember a specific message*?<br>R. "*One message said how important it was to keep taking your pills to keep you fit and healthy*" patient 02028K<br>R. "*one [message] talked about the benefits of taking your medication regularly*" patient 02076E<br>R. "*If I'm honest, no. I've had a holiday in-between. No, I don't – I think probably, the one about – there was one about keeping – you're taking it to keep well, yeah, probably that one, that's the one that sticks in my mind"* patient 01051L<br>Remind to take medications<br>"*For me, the short message was ideal, you know? Who am I? and yes, and, "Have you taken your medication?"* patients 01006W<br>"*I liked the idea of just a phone call to say, "Is that {name}?" "Yeah." "Take your tablet." Straightforward, you know*" patient 01001X<br>"*Oh, well, just the, you know, jogging the memory… Yeah, it's, you know, the telephone rings, somebody comes out with a message, are you so and so? Don't forget to take your tablets and so, you take them*" patient 01025G<br>"*I will say I liked it because it reminded me, like helped me to remember to take my tablet*" patient 01051L<br>"*I thought it was a good system to – it just reminded me to take my tablets*" patient 02028K<br>Social support<br>"*it was interesting to do it and to think someone's taking some notice of what I'm doing, trying to get rid of my high blood pressure*" patient 01024X<br>Habit formation<br>"*Well, the telephone call comes through at the same time every morning and so, you know you're going to take the pills*." Patient 01025G<br>"*Well, I mean, yes, I mean, the phone call alone, without any message at all, would remind you*" patient 01031Q<br>"*It helped the routine, I mean, that was just that reminder of doing things ... by the end I'd developed a bit of a routine that I'm sitting there with the kind of phone ready for {time} o'clock, right, and then I would go and take my tablets*" patient 01057V

Note: Data from patients' (n=17) face-to-face interviews at the end of the one-month pilot intervention (T2).

Participants suggested that the intervention was acceptable and easy to use, and they provided their views about specific features of the intervention delivery mode and content. Specifically, the tailored schedule of the messages, the personalisation, and the variation of the content was

For peer review only - http://bmjopen.bmj.com/site/about/guidelines.xhtml

found to be particularly appealing and were perceived to promote engagement with the intervention (Table 5, Appendix 7).

Table 5. Participants' experiential feedback about the intervention delivery mode.

Theme	Quote
Usability of the intervention, delivery mode, outbound and inbound calls	"*It was certainly easy to use. There's nothing complex about it really. I mean, if you've got any questions, you can just ring the numbers. I didn't have any questions, personally, but if you've got any, you can ring the number and it's quite easy and straightforward*" patient 01006W<br>"*It was always dead on time too, and if I – so, it didn't recognise it, they phoned back, sort of, about five minutes later and then it was alright*" patient 01024X<br>"*Well, it was very easy, just – yeah, very easy. I had clear numbers to call if there was a problem, which I did, on a couple of occasions. No, it was very easy … I think having the choice of time is best… Yes, yeah, no, I think that is important, the choice of time, definitely*" patient 02028K<br>"*It was easy and as long as it was at a set time, which it was, and there's – and if there's a second mobile phone, so if the first one misses, at least you can get hold of the person on the mobile if they're out and about*" patient 01025G

Note: Data from patients' (n=17) face-to-face interviews at the end of the one-month pilot intervention (T2).

Participants also recommended that some messages could be better received if delivered by either IVR or SMS, depending on the timing and the content of the messages. For example, they recommended text messages at the time of medication taking with simple reminders of the behaviour to reinforce habit formation; whereas they recommended voice messages at a time of their preference with information about how to set up a routine with medication taking. They also suggested messages that target patients' INA beliefs and coping plans, to be delivered less frequently than reminders of the behaviour, as they were more personal and therefore easier to be perceived as invasive.

> *"So maybe a text message … 'cause you're waiting for the phone and you've got to do something, which, you know, I think is a good thing, in that you've got to do something and you have to respond. You can't just put your phone in your pocket and walk away, but at night time, I think it would probably be a bit intrusive"* patient 01062T, follow up interview.

Participants reported being satisfied with the overall experience with the intervention, the availability of the intervention 24/7, and their ability to call in and leave a message (see Appendix 8). They would also recommend the intervention to other people who take prescribed medications (see Appendix 7).

For peer review only - http://bmjopen.bmj.com/site/about/guidelines.xhtml

Discussion

Principal Findings

A highly tailored intervention has been developed to support adherence to medication prescribed for high blood pressure and co-morbidities. The intervention has been systematically developed based on guidance from MRC framework, PPI/E and is based on rigorous theory and evidence. It includes a combination of Behavioural Change Techniques (BCTs) tailored to patients' beliefs (e.g., beliefs about medications, self-efficacy, social norms, perceived regimen complexity) and prescription plan. The pilot study suggested that the intervention content increased awareness about the necessity of medication adherence, the risk and benefits of maintaining adherence to prescribed medications, reinforced social support and habit formation, and reminded patients to take medications as prescribed. To our knowledge, this is the first medication adherence intervention delivered using automated text and voice messages that has been developed and piloted within the UK primary care setting. The intervention has been proven to be acceptable and feasible to adults with high blood pressure or co-morbidities, and it is currently being evaluated in a randomised controlled trial.

Strengths and Limitations of this study

To date there are studies describing the systematic development of mHealth interventions, however none of them has provided a detailed description based on guidance from the MRC framework, and none has described the methods used to develop and pilot the implementation procedures within the primary care setting. Furthermore, this study has involved PPI/E in the intervention development, design and pilot, adapting a collaborative approach, which minimized the influence of the social desirability effect on the findings, and thus it provided a real-world perspective. As a consequence, the results of this intervention may be better informed and matched to patients' healthcare needs.

A strength of this study is the systematic approach to intervention development and piloting. The study used a rigorous theoretical approach to guide the development of the tailored intervention content. It also designed a flexible and scalable application to deliver messages to support adherence to different type, and complex prescribed medications. Another strength of this study is the utilisation of a mixed (e.g., qualitative, quantitative) and multi-perspective

For peer review only - http://bmjopen.bmj.com/site/about/guidelines.xhtml

(patients, health care providers, PPI, stakeholders) methods of data collection. The data were combined complementarily into one comprehensive analysis, using a rigorous analytical approach.

Although this intervention is highly tailored, and participants can provide real time input and further tailor intervention content and delivery, a limitation of the intervention is the self-reported theoretical determinants of medication adherence. Future studies could enable the use of objectively measured theoretical determinants and objectively measured behaviour, using sensing technology. Another constrain of the text and voice messaging is its limited features to facilitate delivery of BCTs visually (e.g., picture of tablets, graphs to show levels of adherence) [31], and future studies could usefully explore whether and how these features could influence intervention engagement and behaviour change.

Future Research

The one-month intervention has been extended to three months intervention. It also includes another level of tailoring, where participants select between two BCTs to support maintenance of medication taking (e.g., habit formation or self-monitoring). The intervention is currently being evaluated in a RCT, which includes objective behavioural (medication event monitoring system) and clinical (systolic blood pressure, HbA1c) outcome measurements to inform the results about the efficacy of the intervention (see: https://doi.org/10.1186/ISRCTN10668149).

Funding statement: This paper presents independent research funded by the National Institute for Health Research (NIHR) under its School of Primary Care Research [grant number: RG86506] and the Research for Patient Benefit programme [grant number PB-PG-0215-36032]. The views expressed are those of the authors and not necessarily those of the NHS, the NIHR or the Department of Health. The funder had no role in study design, data collection, data analysis, data interpretation, the writing of the manuscript, and decision to submit the manuscript for publication.

Acknowledgment: This paper presents independent research funded by the National Institute for Health Research (NIHR) under its School of Primary Care Research [grant number: RG86506] and the Research for Patient Benefit programme [grant number PB-PG-0215-36032]. The views expressed are those of the authors and not necessarily those of the NHS, the NIHR or the Department of Health. The funder had no role in study design, data collection, data analysis, data interpretation, the writing of the manuscript, and decision to submit the manuscript for publication.

For peer review only - http://bmjopen.bmj.com/site/about/guidelines.xhtml

INTERVENTION DEVELOPMENT AND PILOTING

Author's contributions: AK conceived and designed the studies, developed the intervention, and led the intervention piloting, data collection, analyses, and wrote this manuscript. VH assisted in data collection. SE and JB provided IT expertise in developing the digital platform. SS provided guidance and consultation to AK during all stages of the intervention development and piloting process.

Conflict of interest: the authors declare no conflict of interest.

Ethics approval and consent to participate: The Office for Research Ethics Committees of Northern Ireland (ORECNI; REC references: 16/NI/0150) and Essex East of England (REC Reference number 17/EE/0203), and the Health Research Authority, have reviewed and approved this project.

Data sharing. Qualitative and quantitative data are available upon request to AK.

References

1. NICE. *Hypertension in adults: diagnosis and management.* Available from: https://www.nice.org.uk/guidance/cg127
2. The Emerging Risk Factors Collaboration. *Association of Cardiometabolic Multimorbidity With Mortality.* Jama, 2015. **314**(1): p. 52-60.
3. Public Health England. *Tackling high blood pressure. From evidence into action*. 2014; Available from: https://www.gov.uk/government/publications/high-blood-pressure-action-plan.
4. Sackett D. *Compliance with therapeutic regimens*. 1976. The John hopkins University. Baltimore & London.
5. Mazzaglia G, Ambrosioni E, Alacqua M, Filippi A, Sessa E, Immordino V, et al., *Adherence to antihypertensive medications and cardiovascular morbidity among newly diagnosed hypertensive patients.* Circulation, 2009. **120**(16): p. 1598-605.
6. Chowdhury R, Khan H, Heydon E, Shroufi A, Fahimi S, Moore C, et al. *Adherence to cardiovascular therapy: a meta-analysis of prevalence and clinical consequences.* Eur Heart J, 2013. **34**(38): p. 2940-8.
7. Cassell A, Edwards D, Harshfield A, Rhodes K, Brimicombe J, Payne R, et al. The epidemiology of multimorbidity in primary care: a retrospective cohort study. Br J Gen Pract, 2018.

For peer review only - http://bmjopen.bmj.com/site/about/guidelines.xhtml

8. NCD Risk Factor Collaboration. *Worldwide trends in blood pressure from 1975 to 2015: a pooled analysis of 1479 population-based measurement studies with 19.1 million participants.* Lancet, 2017. **389**(10064): p. 37-55.
9. NICE. *Medicines Optimisation: The Safe and Effective Use of Medicines to Enable the Best Possible Outcomes*. 2015, National Institute for Health and Care Excellence (UK)
10. NICE. *Medicines Adherence: Involving Patients in Decisions About Prescribed Medicines and Supporting Adherence*. 2009, Royal College of General Practitioners (UK)
11. Tsoli S, Sutton S, Kassavou A. *Interactive voice response interventions targeting behaviour change: a systematic literature review with meta-analysis and meta-regression* BMJ Open 2017;0:e018974. doi:10.1136/bmjopen-2017-018974
12. Kassavou A, Sutton S. *Automated telecommunication interventions to promote adherence to cardio-metabolic medications: meta-analysis of effectiveness and meta-regression of behaviour change techniques.* Health Psychol Rev, 2017: p. 1-18. doi: 10.1080/17437199.2017.1365617.
13. Smith DH, O'Keefee-Rosetti M, Owen-Smith AA, Rand C, Tom J, Vupputuri S, et al. *Improving Adherence to Cardiovascular Therapies: An Economic Evaluation of a Randomized Pragmatic Trial.* Value Health, 2016. **19**(2): p. 176-84.
14. Burn E, Nghiem S, Jan S, Redfern J, Rodgers A, Thiagalingam A, et al. *Cost-effectiveness of a text message programme for the preventin of recurrent cardiovascular events*. Heart, 2017. **103**: p. 923-930.
15. Yardley L, Morrison L, Bradbury K, Muller I. *The person-based approach to intervention development: application to digital health-related behavior change interventions.* J Med Internet Res, 2015. **17**(1): p. e30.
16. Mummah SA, King AC, Gardner CD, Sutton S. *Iterative development of Vegethon: a theory-based mobile app intervention to increase vegetable consumption.* Int J Behav Nutr Phys Act, 2016. **13**: p. 90.
17. Michie S, Atkins L, West R. *The Behaviour Change Wheel. The guide to developing interventions.* 2014, London

For peer review only

For peer review only - http://bmjopen.bmj.com/site/about/guidelines.xhtml

18. Craig P, Dieppe P, Macintyre S, Michie S, Nazareth I, Petticrew M, et al., Developing and evaluating complex interventions: the new Medical Research Council guidance. Bmj, 2008. 337: p. a1655.

19. Dijkstra A, De Vries H. *The development of computer-generated tailored interventions.* Patient Educ Couns, 1999. **36**(2): p. 193-203.

20. Kreuter MW, Farrell D, Olevitch L, Brennnan L. *Tailoring Health Messages. Customizing communication with computer technology*. Lawrence Erlbaum Assoc, 1999.

21. Barber N. *Should we consider non-compliance a medical error?* Qual Saf Health Care, 2002. **11**(1): p. 81-4.

22. Kassavou A, Sutton S. *Developing and pre-testing a tailored interactive voice response (IVR) intervention to support adherence to anti-hypertensive medications.* 2016, research protocol, The University of Cambridge.

23. Michie S, Richardson M, Johnston M, Abraham C, Francis J, Hardeman W, et al., *The behaviour change technique taxonomy (v1) of 93 hierarchically clustered techniques: building an international consensus for the reporting of behaviour change interventions.* Ann Behav Med, 2013. **46**(1): p. 81-95.

24. Farmer A, Hardeman W, Hughes D, Prevost AT, Kim Y, Craven A, et al. *An explanatory randomised controlled trial of a nurse-led, consultation-based intervention to support patients with adherence to taking glucose lowering medication for type 2 diabetes.* BMC Fam Pract, 2012. **13**: p. 30.

25. Kassavou A, Sutton S. *Reasons for non-adherence to cardiometabolic medications, and acceptability of an interactive voice response intervention in patients with hypertension and type 2 diabetes in primary care: a qualitative study.* BMJ Open, 2017. **7**(8): p. e015597.

26. Wilkinson S. *Qualitative data collection: interviews and focus groups*, in *Research methods for clinical and health psychology*, Yardley, L., Marks D, Editor. 2004. Sage Publications Ltd.

27. Kassavou, A., Houghton, V., Edwards, S., Brimicombe, J., Wilson, E., Griffin, S., Sutton, S. (2018). *Acceptability of the Medication Adherence for Patients Support (MAPS) intervention to improve adherence to patients prescribed multiple medications, as an*

For peer review only

adjunct to primary care: a qualitative study. Accepted for publication in Journal of Health Psychology.

28. Alexander V, Thomas H, Cronin A, Fielding J, Moran-Ellis J. *Mixed methods* in *Researching Social Life*, N. Stoneman, Editor. 2016: London p. 119-138.
29. Moran-Ellis J. *Following a Thread – An Approach to Integrating Multi-method Data Set.'* Paper given at ESRC Research Methods Programme, Methods Festival Conference, Oxford, July 2004.
30. O' Cathain A, Murphy E, Nicholl J. Three techniques for integrating data in mixed methods studies. BMJ, 2010; 341:c4587.
31. Armitage L, Kassavou A, Sutton S. A systematic review of the literature with meta analysis and meta regression: the use of smartphone applicatons to suport medication adherence. Prospero protocol, 2018.

Appendix 1. Intervention development process, informed by the MRC Framework

Appendix 2. Intervention piloting process, informed by the MRC framework

Appendix 3: Map of the theoretical framework, determinants and BCTs to produce changes in adherence to medications.

Appendix 4: PPI/E messages

Appendix 5: Example of message tailoring.

Appendix 6. Participants' demographic characteristics

Appendix 7. Participants' perception of the intervention utility and future use

Appendix 8. Participants' satisfaction with the intervention components.

For peer review only

Appendix 1. Intervention development process, informed by the MRC Framework.

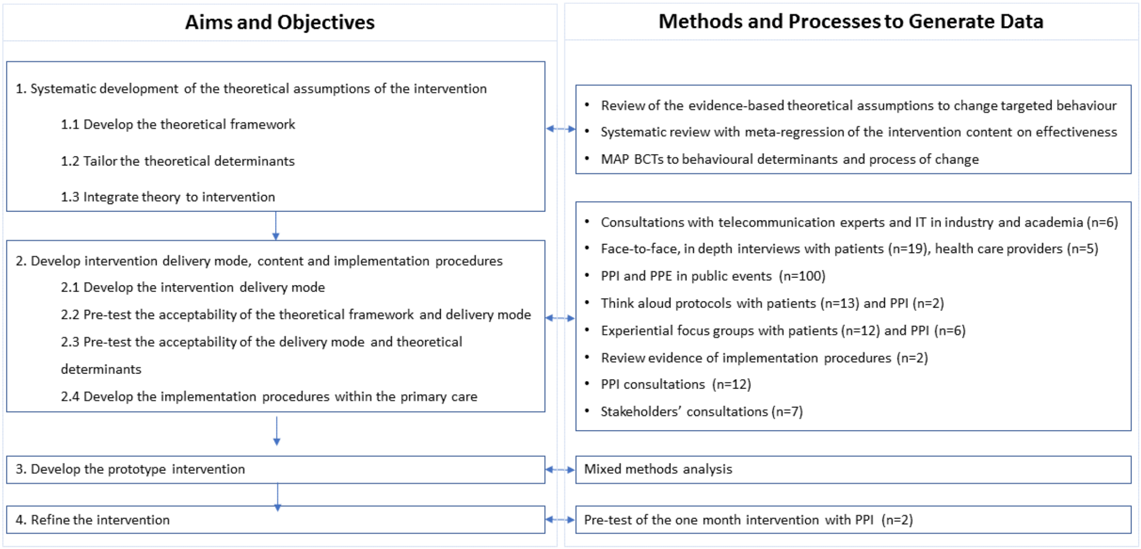

Appendix 2. Intervention piloting process, informed by the MRC framework.

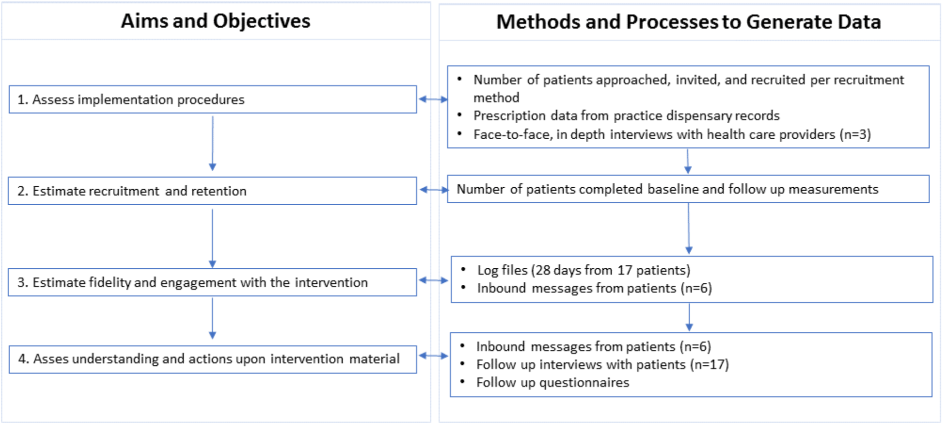

For peer review only

Appendix 3. Map of the BCTs to theoretical determinants and framework.

	Framework	Theoretical Determinant	BCTs
PRIMARY DETERMINANTS	INA	Beliefs and knowledge	*Re-attribution*
			Pros and cons
			Information about antecedents
			social comparison
			reduce negative emotions
			normative information
		outcome expectations	*Information about health consequences*
			information about emotional consequences
			Information about social and environmental consequences
			Pros and cons
			goal setting (behaviour)
			goal setting (outcome)
			self talk
			Social support (unspecified)
			Social support (practical)
			Socia support (emotional)
		self efficacy	*feedback on behaviour*
			reporting whether or not the behaviour was performed
			Problem solving
			action planning/ implementation intentions
			self monitoring of behaviour
			adding objects to the environment
			feedback on outcomes of behaviour
			biofeedback
			review behaviour goals
	NINA		*review outcome goals*
			feedback on emotional state
			monitoring of emotional consequences
			social reward
			Social support (unspecified)
			Social support (practical)
			Socia support (emotional)
			self talk
		habit	*Prompts/cues*
			habit formation
			action planning/ implementation intentions
			feedback on behaviour
			Social reward
SECONDARY DETERMINANTS		social support	*social comparison*
			social reward
			Social support (unspecified)
			Social support (practical)
			Socia support (emotional)
		emotional/ affective state	*Information about emotional consequences*
			Report emotional state
			Feedback on emotional state
			Monitoring of emotional consequences

Appendix 4. Examples of Patient and Public Involvement and Engagement (PPI/E) messages.

Scenario: Mark is a 49 year old car mechanic. He was diagnosed with high blood pressure by his GP 6 months ago even though he had no symptoms, was not overweight, did not smoke, ate healthily and had an active job. Initially, he took the medication exactly as his GP prescribed every day. However, after a few weeks he began to experience constipation and read on the leaflet supplied with the tablets that this was a side effect caused by the medication. Now, he only takes his medication every other day and the constipation has cleared up. However, his blood pressure is still too high.

**Message**	**Event**
Keep on the tablets prescribed to you by your Doctor. High blood pressure is more serious than constipation.	HOP
His action was foolish – he should have consulted his GP before changing the prescribed medication.	HOP
This scenario similar to mine. However I do take my meds without fail. I am motivated by my family history which leads me to believe I may be susceptible to heart disease which, of course, is thought to be related to blood pressure. I can understand why people might be concerned about side effects, so they need motivation.	HOP
I know that mechanics are frequently asked to work through lunch breaks etc. Please take some fruit and water to work with you to snack on throughout the day. This will help with your constipation, as you should be taking your pills every day. Good luck.	HOP
You need to talk to your GP in order for him to prescribe a laxative to help with the constipation. The need to take your BP medication is crucial in bringing your BP down.	HOP
BP tablets must be taken every day so consult your GP for medication for constipation.	HOP
It's stupid not to take your meds! If you're constipated just eat more fibre. It's not worth the risk.	HOP
Hey Mark. Are you aware of your blood pressure today? Have you taken your meds yet?	SF
Hi Mark. I sympathise a lot. I used to suffer from blood pressure and constipation, but nowadays I rarely suffer from either. I've had heart surgery but still cycle a lot. Don't give up on the meds. There are different tablets available: ask your GP. But also, use whole brand cereals regularly, even at bedtime!	SF
Hi Mark. You've sorted the constipation – keep going for the blood pressure!	SF
Mark, you should keep taking your medication even with the side effects, because it is better for you.	SF

Abbreviations: Hospital Open Event – HOP; Science Festival – SF; Focus Groups – FG

For peer review only

Appendix 5. Examples of tailoring messages.

Tailored theoretical determinants	BCT	Message file
↑ NINA and ↑ emotional state	Habit formation	Please do not forget to take your tablets. To achieve better control over high blood pressure, you will need to take them every day.
NINA / INA and ↓ social norms	Report whether or not the behaviour was performed, social reward, and information about health consequences	IVR: We are calling to see how you are doing with taking your tablets. Have you taken all your tablets as prescribed today?<br>P: yes<br>IVR: Well done! Taking your medications as prescribed is an easy way to keep blood pressure down.
↑ NINA	Habit formation	You are soon due to take your tablet. Please do not forget to do so.
NINA / INA and prescription plan	Habit formation and action planning	This is your message for your blood pressure tablets. One easy way to remember your tablets, is to take them with another daily activity, such as your morning cup of tea. If they are always done together, it will be harder to forget.
↑ Beliefs about medications, ↑ self-efficacy to take medications when no health benefits and ↑ emotional state	Habit formation and information about emotional consequences	Please keep taking your tablets as prescribed even if you are well and feeling healthy. High blood pressure is one of those things that unless you actually feel it you're not aware that it is a problem. Taking your medications as prescribed will support you to keep enjoying things or activities that are important to you.
NINA, ↓ beliefs about medications and ↓ emotional state	Habit formation and social support (unspecified)	Whatever the day may holds, please do not forget to take your tablets. To achieve better control over high blood pressure, you will need to take them every day.
↓ self-efficacy to cope with ↓ practical social support	Habit formation and social support (unspecified)	We are calling to see how you are doing with taking your tablets today. Please remember that you can always call the free telephone number if you require more support on how to take your tablets as prescribed. If you require more information about the health condition or the dosage of your medications, please contact your GP practice or pharmacist.
↑ social norms and ↑ self-efficacy to cope with ↓ practical social support	Habit formation and social support (unspecified)	You may find it helpful to ask people who are important to you to remind you to take your medications as prescribed and maintain your blood pressure under control. Please remember that you can always call the free telephone number if you require more support on how to take your tablets as prescribed.

Note: ↑indicate higher score from the cut-off point, ↓ indicate lower score from cut-off point.

For peer review only - http://bmjopen.bmj.com/site/about/guidelines.xhtml

Appendix 6. Participants' demographic characteristics

Age		Work status		Health condition		Gender	
80-89	7.8%	Retired	76.5%	Hypertension	64%	Male	65.7%
70-79	28.1%	Part-time	10.9%	Type 2 Diabetes	14%	Female	34.3%
60-69	43.7%	Full time	10.9%	Co-morbidities	22%		
50-29	15.6%	Unable to work	1.7%				
40-49	4.6%						

Note: results from N=64 patients taking part in intervention development studies (interviews n=19; think aloud protocols n=13; focus groups n=12; consultations n=2) and pilot study (n=18).

For peer review only

Appendix 7. Participants' perception of the intervention utility and future use

	Mean (sd)
The intervention messages helped me to take my tablets as prescribed	3.2 (1.3)
The intervention reminded me to take my medications as prescribed	3.5 (1.3)
The intervention was easy to use	4.3 (0.7)
The intervention addressed my personal needs	3.3 (1.1)
It would have been useful, if the intervention was part of the regular care	3.7 (0.9)
I would recommend the intervention to other people who take medications for long-term health conditions	4.2 (0.8)

Note: Likert scale, 1 strongly disagree to 5 strongly agree. Data from n=17 participants.

For peer review only

Appendix 8. Participants' satisfaction with the intervention components.

	Mean (sd)
The experience with the intervention	4 (0.7)
The voice delivering the intervention messages	3.7 (1.1)
The content of the intervention messages	3.8 (0.8)
Being able to call the intervention	4 (0.7)
The availability of the intervention 24/7	4.2 (0.8)
The response you received from the intervention	3.4 (0.7)
The response you received to your particular queries/questions	3.4 (0.7)

Note: Likert scale, 1 very dissatisfied to 5 very satisfied. Data from n=17 participants.

For peer review only
